# ⁷Li NMR short-range ordering in hardened lithium sodium niobate

**DOI:** 10.1038/s41598-025-21740-w

**Published:** 2025-10-09

**Authors:** Millena Logrado, Anuraag Gaddam, Fangping Zhuo, Changhao Zhao, Shuang Gao, Hergen Breitzke, Markus Rosenstihl, Michael Vogel, Jürgen Rödel, Gerd Buntkowsky

**Affiliations:** 1 Department of Chemistry, Eduard-Zintl Institute for Inorganic and Physical Chemistry, Technical University of Darmstadt, 64289 Darmstadt, Germany; 2 Institute of Physics of São Carlos, University of São Paulo, São Carlos, São Paulo 13566-590 Brazil; 3 Department of Chemistry, CICECO – Aveiro Institute of Materials, University of Aveiro, Aveiro, Portugal; 4 Division of Nonmetallic-Inorganic Materials, Department of Materials and Earth Sciences, Technical University of Darmstadt, 64289 Darmstadt, Germany; 5 State Key Laboratory of Electrical Insulation and Power Equipment, School of Electrical Engineering, Xi’an Jiaotong University, Xi’an, 710049 Shaanxi People’s Republic of China; 6 Key Laboratory of Green Extraction & Efficient Utilization of Light Rare-Earth Resources (Inner Mongolia University of Science and Technology), Ministry of Education, School of Rare Earth Industry, Baotou, 014010 People’s Republic of China; 7 Institute for Condensed Matter Physics, Technical University of Darmstadt, 64289 Darmstadt, Germany

**Keywords:** NMR, DFT, Lithium niobate, LiNb_3_O_8_, LNN ceramics, Precipitates, Chemistry, Materials science, Physics

## Abstract

Thermal treatments play a crucial role in tuning the functional properties of lithium-based perovskites, which are of interest for applications in energy storage, actuation and sensing. In the present work, the impact of aging and quenching on the local structure and lithium mobility in lithium sodium niobate (LNN) ceramics was investigated using Density Functional Theory (DFT), transmission electron microscopy (TEM) and solid-state Nuclear Magnetic Resonance (NMR) techniques. Our results demonstrate that quenching inhibits the formation of a lithium-rich phase, leading to significant modifications in dipolar and quadrupolar interactions. Aging induces the formation of LiNbO_3_ within the perovskite matrix, with no indication of lithium mobility at the boundaries of LiNbO_3_ grains. The presence of multiple lithium environments in aged samples was indicated by transmission electron microscopy and confirmed through ^7^Li Magic Angle Spinning (MAS) NMR, revealing distinct structural differences between aged and unaged samples. NMR parameters were also compared with parameters calculated by Density Functional Theory. The findings suggest the formation of LiNbO_3_ and an additional lithium poor phase in aged LNN samples. These results indicate that the quenching effect reduces dipolar interactions and considerably sharpens the ^7^Li NMR spectra, while structural modifications induced by thermal processing affect lithium ordering, providing new insights into the phase behavior of LNN ceramics.

**Supplementary Information:**

The online version contains supplementary material available at 10.1038/s41598-025-21740-w.

## Introduction

Lead-free piezoceramics are promising substitution materials for problematic lead-containing electric and electronic materials^1–3^. Thermal treatments of these materials, such as quenching and aging, significantly alter the local structure and improve the electrical properties of functional ceramics^4–9^. Quenching, through rapid cooling, preserves high-temperature defect structures, impacting phase stability and electrical conductivity. In contrast, aging allows to establish an equilibrium phase structure and enables defect reorganization, restoring resistivity and modifying microstructure.

Multiple studies show that quenching ceramics significantly increases the number of oxygen vacancies^4,6–8^. In particular, the interfacial regions of nanocrystalline LiNbO_3_ grains appear to be strongly affected by their presence^10^. These interfacial regions seem to serve as a reservoir for fast-diffusing Li ions, with ion exchange mediated by their close proximity to oxygen vacancy defects, while the bulk exhibits lower ion mobility^11–13^. The reported diffusion parameters are frequently related to fine-tuning of engineering interface material, with grain sizes up to 50 nm. Similar effects have also been reported–in contrast to the behavior observed in nanograins—in samples with much larger grain sizes, presenting a distribution ranging from 4 to 500 nm with a predominance of larger particles^14^. In contrast to quenching, which increases oxygen vacancies, specific heating treatments can reduce their concentration by promoting the oxidation of the ceramic^4,6,8,12^. The combination of these effects can be used to tune specific properties. Moreover, recent NMR studies on argyrodite-type solid electrolytes highlight how both bulk and grain boundary regions critically govern Li-ion dynamics, showing that the interplay between crystal structure, interfaces, and defects directly impacts ionic conductivity^15–17^.

For instance, aging steps were used to form a secondary phase in ferroelectric ceramics to promote for precipitation hardening^5,18–21^. In the recent work of Zhao et al.^5,18,22^, the main objective of quenching is to freeze the high-temperature single-phase structure and prevent the formation of secondary phases, whereas aging promotes the controlled nucleation and growth of LiNbO_3_ secondary phase in LNN to help to pin domain walls and induce a hardening effect. Furthermore, a detailed study using TEM on the topology of LiNbO_3_ precipitates formed in LNN ceramics was published^22^, revealing a dense lamellar contrast inside the precipitate itself, but no explanation was provided for this finding.

Precipitation is a process technology predominantly employed in metals to impart increased hardness and fracture strength^23^. In ceramics it traditionally has only been employed to toughen zirconia materials by inserting a metastable tetragonal phase. Only recently, ferroelectric hardening was introduced. The delocalized electron gas in metals does not allow for effective local investigation of the evolution of local structure. Using such techniques in ceramics with the intricate local structure opens now a pathway to investigate the evolution of precipitation in considerably more resolution^23^.

To investigate this dense lamellar contrast, a short-range order investigation using ^7^Li NMR was employed. NMR is a powerful technique for studying (a) ^7^Li ion mass hopping—including the ^7^Li exchange, when two ions swap positions rather than moving independently—and (b) local structure, whether ordered or disordered, due to its ability to analyze samples without requiring translational long-range order^11–14,24–27^. In this work, we focus on the short-range order of ^7^Li in Li_0.18_Na_0.82_NbO_3_(LNN18). We investigate the impact of quenching and thermal processing on lithium environments within the perovskite matrix. The study explores possible LiNbO_3_ mobility as well as the formation of secondary LiNbO_3_ phases and potential lithium-poor regions in aged samples. High-resolution solid-state NMR is employed to probe distinct ^7^Li sites, complemented by Density Functional Theory calculations and transmission electron microscopy to provide structural insights.

## Results

Samples analyzed in this study are listed in Table 1. For more information about the samples, please check the Material and Methods section. The Results section is organized into two parts. First, we present NMR data on the LNN18 sample to investigate the presence or absence of slow and fast lithium species, thereby assessing ionic mobility. LiNbO_3_ nanocrystals, as well as LiNbO_3_-containing ceramics and glasses often exhibit ionic mobility^11–14,28^, which has a strong influence on its NMR spectra. In this study, we present compelling evidence of the lack of ionic mobility for the ceramics examined in the first part. Instead, all the features observed in the spectra were interpreted as structural features. This interpretation is further supported by the results presented in the second part and counts with TEM images and high-resolution ^7^Li NMR spectra, along with the extracted nuclear parameters, such as the isotropic chemical shift and quadrupolar coupling constant. DFT calculations are presented to validate NMR results and compare different crystalline phases.

**Table 1:** Systhesis of thermal treatment of samples 18 LiNbO_3_ – 82 NaNbO_3_ (LNN18).

Pe-U18	Pellet sample sintered at 1300 °C for 2 h, heat treated at 1300 °C, air quenching: Unaged LNN18 sample
Pe-A18	Pellet sample sintered at 1300 °C for 2 h, heat treated at 1300 °C, air quenching. Heat treated at 500 °C for 24 h, followed by a second heat treatment at 600 °C for 8 h: Aged LNN18 sample
Po-U18	Powder sample sintered at 1300 °C for 2 h, heat treated at 1300 °C, air quenching: Unaged LNN18 sample
Po-A18	Powder sample sintered at 1300 °C for 2 h, heat treated at 1300 °C, air quenching. Heat treated at 500 °C for 24 h, followed by a second heat treatment at 600 °C for 8 h: Aged LNN18 sample

### Mobility

**Fig. 1:**
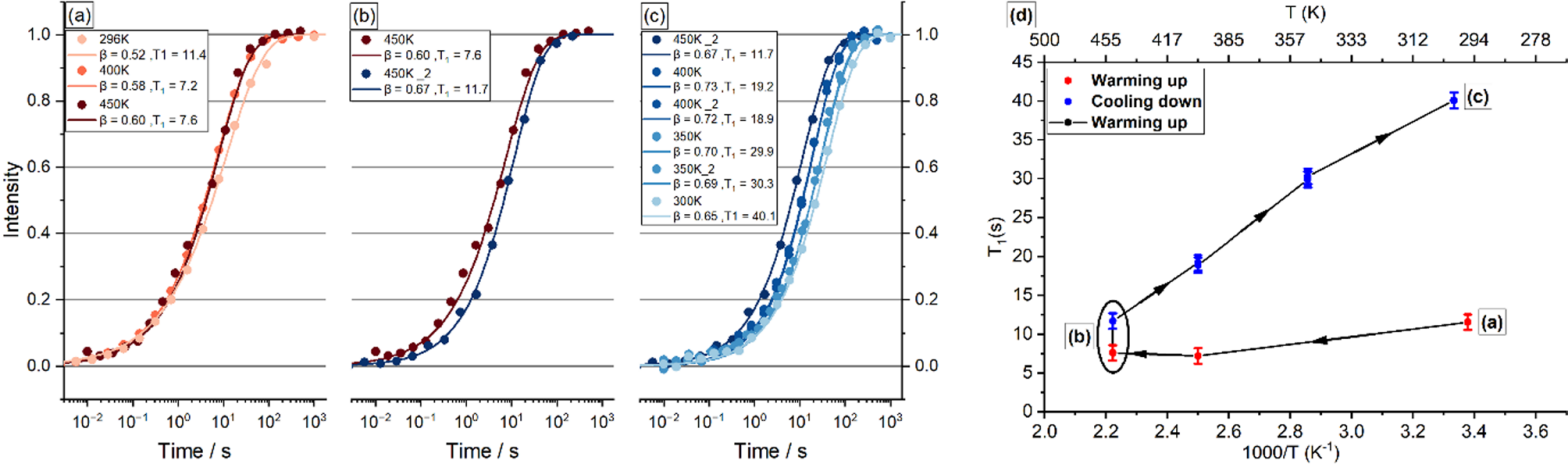
(**a**), (**b**) and (**c**) present T_1_ measurements at different temperatures ranging from 296 to 450 K. In (**a**), T_1_ curves are depicted during the heating process. In (**b**), two T_1_ measurements at 450 K reveal distinct values, indicating changes in the network under this condition, above in text interpreted as phase transition. In (**c**), the system was cooled from 450 to 300 K. Heating and cooling down processes were performed sequentially. In (**d**), the T_1_ vs. temperature experiments are summarized.

To investigate mobility, spin–lattice relaxation behavior as a function of temperature was studied. Figure 1 depicts results during four distinct heating or cooling processes, highlighting the temperature dependence of relaxation. In Fig. 1a, the Pe-A18 was heated from 296 to 450 K. The data points were fitted using Eq. (2), allowing the determination of the longitudinal relaxation time (T_1_) and the stretching parameter (β). In (b), the experiment at 450 K was repeated in sequence under identical conditions. Each experiment took about 24 h to complete. Although no changes were made in the experimental parameters, a noticeable difference in T_1_ was observed in the process (b).

Figure S1 (top) shows all the ^7^Li static spectra acquired in processes (a) and (c) illustrating that neither sharpening of the central peak nor motional narrowing was observed within this temperature range. Figure S1 (bottom, right) confirms that the Full Width at Half Maximum (FWHM) of the central peaks remains unchanged throughout the temperature ranges of processes (a) and (c). Figure S1 (bottom, left) presents a supplementary activation energy (E_a_) calculation extracted from T₁ relaxation using the Bloembergen–Purcell–Pound (BPP) theory in the low-temperature regime. Overall, after the first heating, T_1_ shows a temperature dependence described by an activation energy of E_a_ = 0.04 eV, while the stretching parameter β exhibits values around 0.6 in all measurements, which indicates a nonexponential decay due to structural and dynamical disorder^29–31^.

Figure S2 (top) depicts, on the left, the sample inside the sample holder before stage (a) and, on the right, after the full cooling down, at the end of stage (b). The clear change in color provides strong evidence of a phase transition. Figure S2 (bottom), presents the ^23^Na spectra of the central peak of aged LNN18, recorded before heating and immediately after the cooling process. Spectra with very distinct peaks indicated distinct crystalline structure. As further discussed, the change in color, T1 behavior and presence of distinct ^23^Na environments confirms the occurrence of a irreversible phase transition, illustrating that a new structure was formed after the transition, no longer corresponding to Pe-A18.

Jumping rates corresponding to correlation times significantly longer than 100 µs are ineffective on the NMR timescale and, therefore, do not alter the spectral lineshape. To investigate the possible presence of slow-moving ^7^Li—characterized by a jump rate between milliseconds and seconds—Stimulated Echo Decay (SED) measurements were performed in sample Pe-A18. For this experiment, a new Pe-A18 sample was used, which had not undergone the phase transition observed in the previous experiment. Maximum temperature was limited to 450 K to avoid structural changes in the crystal lattice. Even though the total running time for each SED was set to approximately 36 h, the resulting decay curves were still very noisy due to the long relaxation delays of ^7^Li (e.g. see Figure S3a).

**Fig. 2:**
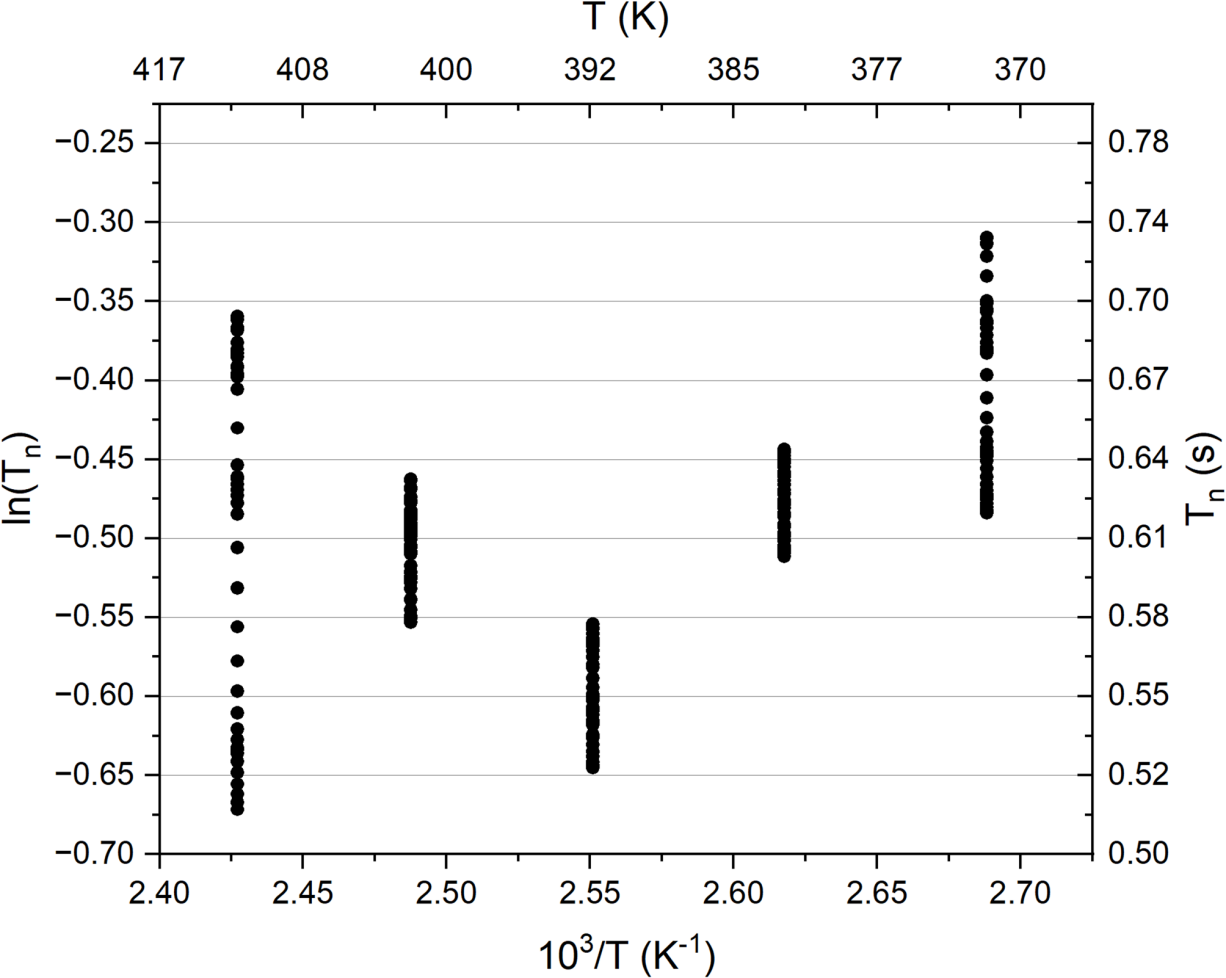
Natural logarithm of *T*_*n*_ plotted against the inverse of temperature. *T*_*n*_ was extracted from Eq. (1)—used fitted the SED curves of aged LNN18 samples.

The echo intensity was determined by integrating the signal within a time window centered at the theoretically predicted point of maximum intensity (see time window delimited by dashed lines in Figure S3a). However, calculating the echo intensity by integrating the signal over a single time window may introduce significant errors. To mitigate this, we estimated the experimental error by integrating within a symmetric range [*‒ n*, *n*] around the theoretical maximum. The parameter *n* defines the number of points included on each side of the maximum intensity (see Figure S3b and c). For each distinct value of *n*, the corresponding echo integration was calculated and plotted against *t*_m_—as represented in Figure S3b and c—and fitted to Eq. (1). The value of n ranged from 20 to 90, yielding 36 distinct points. Figure 2 illustrates the result of the inverse of the temperature against logarithm of relaxation times *T*_*n*_ for each *n*. No temperature dependence is observed; therefore, no attempt was made to fit an Arrhenius equation, as the data show only random fluctuations rather than a linear trend observed when the lithium ionic jumps are present.

#### Structural environments

**Fig. 3:**
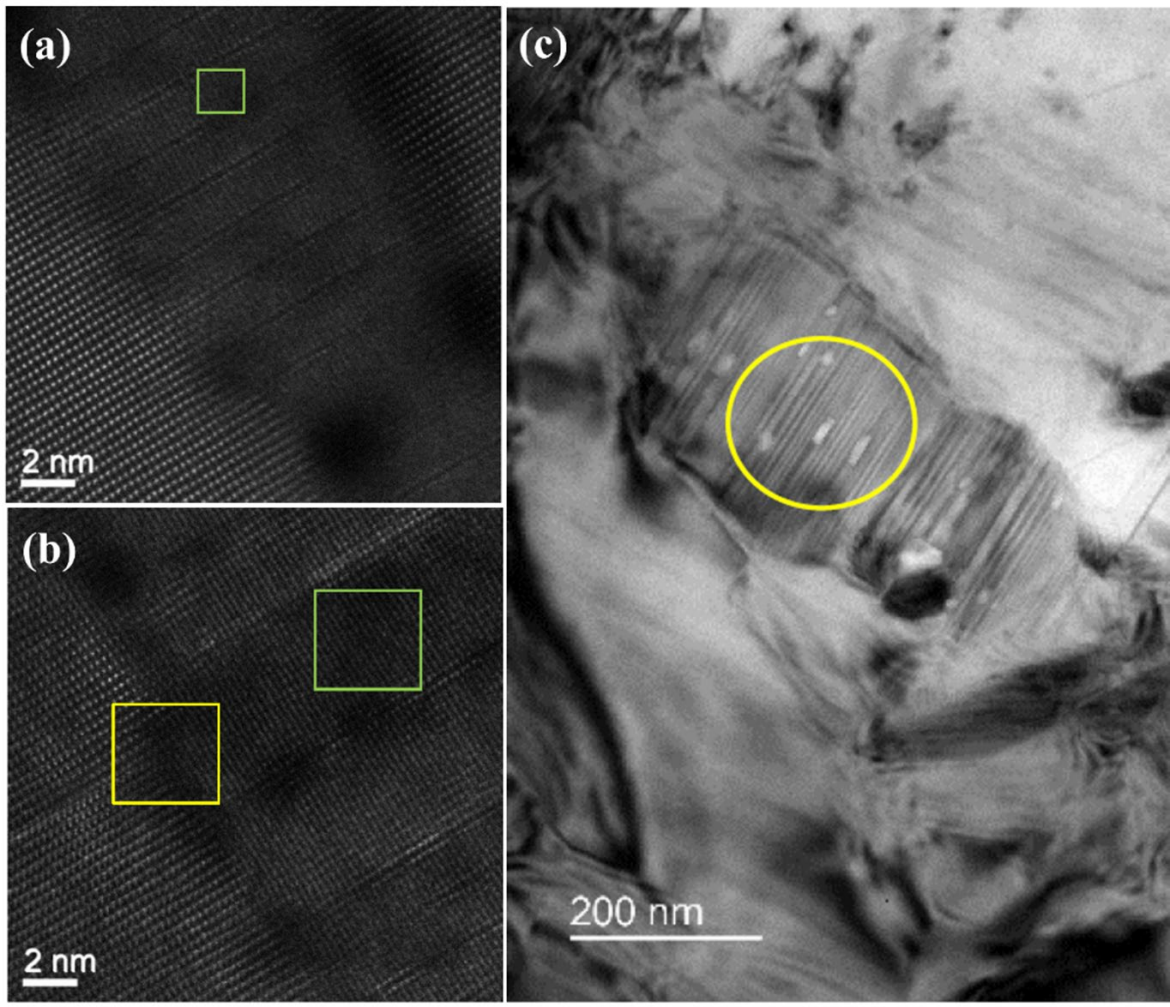
(**a**), (**b**) High-resolution STEM images highlighting contrast inside the LiNbO_3_. The green boxes mark the areas within the precipitate, while the yellow box indicates the interface between the matrix and the precipitate. (**c**) A representative Brigh-field STEM image taken from the Pe-A18 sample. Lamellar structures can be found in the area marked with yellow ellipse.

To investigate the presence of precipitates in aged samples, TEM images of the Pe-A18 sample were collected to gain insights into the local morphology and phase composition. Although TEM is relatively insensitive to lithium due to its low atomic number, contrast variations within the LiNbO_3_ matrix—possibly arising from distinct structural ordering—were still observed, as presented in Figs. 3a and b. Among these features, lamellar structures characterized by alternating light and dark bands were identified, as illustrated in Fig. 3c. However, achieving atomic-scale visualization of lithium atoms together with heavier elements remains challenging for conventional STEM techniques such as annular bright field imaging, differential phase contrast, or single-slice ptychography. To overcome these limitations, advanced methods like multi-slice electron ptychography have been developed^32^, which iteratively reconstruct both the probe and the object through the sample thickness, thereby enabling high lateral resolution along with depth-resolved structural information. Alternatively, complementary techniques such as NMR can be employed to probe the local environment of lithium atoms at the atomic level, offering valuable insights into lithium distribution that may not be accessible via electron microscopy alone.

**Fig. 4:**
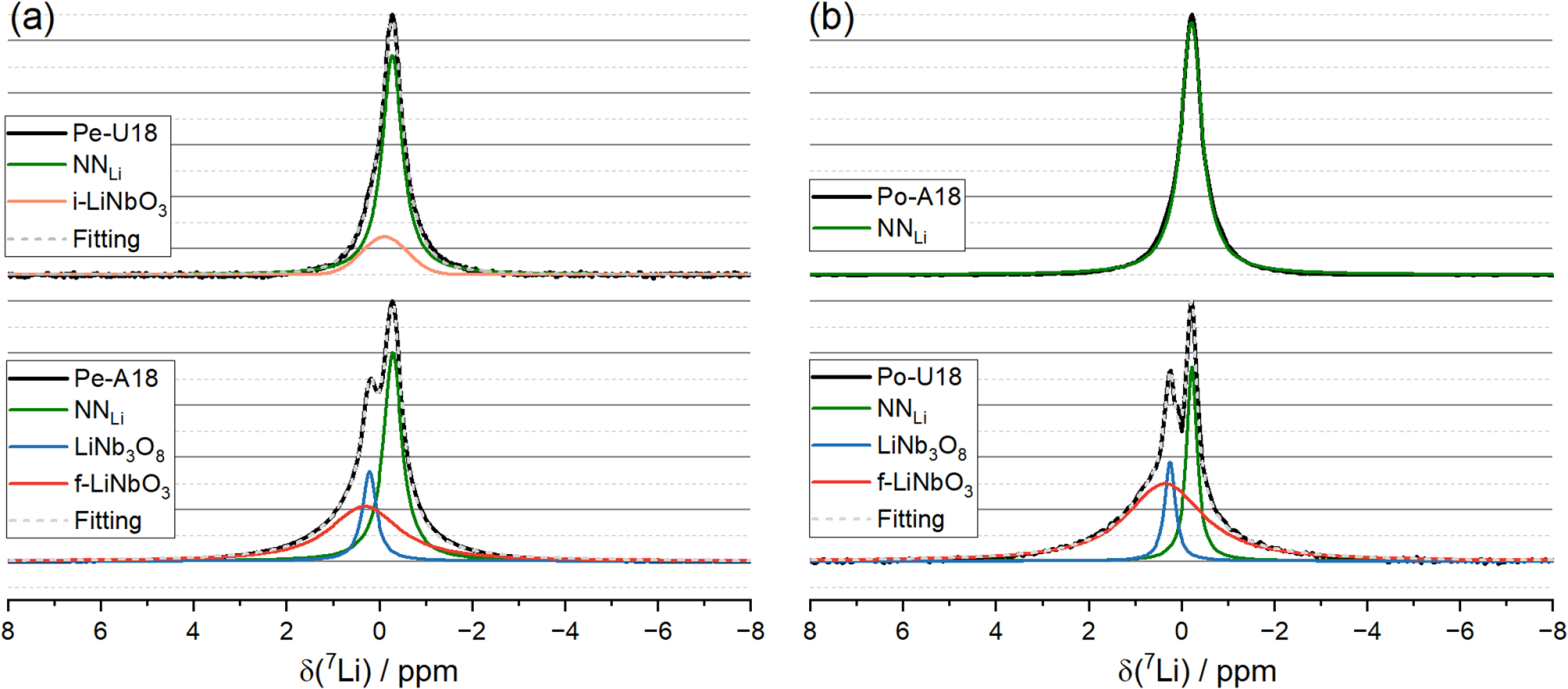
(**a**) ^7^Li spectra of Pe-U18 (top) and Pe-A18 (bottom) samples at 10 kHz, respectively. (**b**): ^7^Li spectra of Po-U18 (top) and Po-A18 (bottom) samples at 10 kHz, respectively.

Structural environments are best studied using high-resolution NMR to eliminate anisotropic interactions that can hide details in the local atomic arrangement. By minimizing anisotropic effects, high-resolution NMR enables differentiation of the electronic environments at distinct sites, as illustrated in Fig. 4. The notation of distinct environments is presented in Table 2, and the results supporting this assignment are discussed in the Discussion section. Figure 4a displays the central peak of the ^7^Li spectra for the pellets. The spectra of the unaged samples are exhibited on top, and the aged samples on the bottom. Figure 4b demonstrates the same information but for powder samples. Both aged samples (Pe-A18 and Po-A18) exhibit very similar behavior, and their spectra were deconvoluted into two sharper components, NN_Li_ and LiNb_3_O_8_, and one broader component, f-LiNbO_3_. The spectrum of the Pe-U18 sample appears slightly broader compared to that of Po-U18; therefore, the first was simulated as a combination of NN_Li_ and i-LiNbO_3_, while the latter was modeled as pure NN_Li_. The interpretation of each of these sites is provided in the Discussion section. Table 3 summarizes the parameters used in each spectral simulation. After normalizing of the maximum spectral intensity to 1, the Root Mean Square Deviation of the fits of the central peaks was found to be above 0.012, indicating that the average deviation between experimental and fitted curves was, approximetely, 1.2% of the maximum signal.

**Table 2:** Nomenclature of sites observed in aged and unaged 18 LiNbO_3_–82 NaNbO_3_ (LNN18) samples.

NN_Li_	Li dispersed in the sodium-niobium (NN) matrix
i-LiNbO_3_	Incipient LiNbO_3_ nucleation
f-LiNbO_3_	Fully formed of LiNbO_3_ precipitates
LiNb_3_O_8_	LiNb_3_O_8_—lithium-poor phase from LiO_2_–NbO_3_ phase diagram

**Table 3:** ^7^Li NMR parameters were extracted using Voigt function: isotropic chemical shift (δ_iso_), corresponding Lorentzian (L) and Gaussian (G) parameters used in the Voigt function.

Samples		Phase	δ_iso_/ppm (± 0.05)	L (Hz) (± 20)	G(Hz) (± 40)
Pe-	U18	NN_Li_	− 0.28	123	0
		i-LiNbO_3_	− 0.01	0	273
	A18	NN_Li_	− 0.29	111	0
		LiNb_3_O_8_	0.22	83	0
		f-LiNbO_3_	0.32	400	100
Po-	U18	NN_Li_	− 0.21	134	0
	A18	NN_Li_	− 0.22	62	0
		LiNb_3_O_8_	0.25	62	0
		f-LiNbO_3_	0.38	449	100

The site named as “LiNb_3_O_8_” proved challenging to identify: XRD did not reveal a third site in the aged samples^19^, and TEM provided only indirect evidence of its presence in Pe-A18^18^. Consequently, complementary analyses combining NMR measurements on Pe-A18 and DFT calculations were undertaken to resolve the three distinct local environments in the aged material. Therefore, a complementary analysis combining NMR on Pe-A18 and DFT calculations were employed to distinguish the three distinct local environments in the aged samples. This analysis aims to provide insight into a possible correlation between the third site observed by NMR in the aged samples and the lamellar structure revealed by TEM in Pe-A18. Given the clearer combined evidence in Pe-A18, this sample was chosen for the following experiments.

In NMR, quadrupolar interaction manifests in satellite transitions, which contribute to the intensities of the spinning sideband (SSB) patterns in MAS NMR experiments. To simulate the SSB pattern, quadrupolar, dipolar, and chemical shift anisotropy interactions must be considered. However, as ^7^Li in Na_*x*_Li_1-*x*_NbO_3_ structures exhibit low chemical shift anisotropy values as evidenced by literature and DFT calculations presented above^33^, chemical shift anisotropy was neglected. Additionally, a second approximation in the simulation considered only quadrupolar interaction for simulation the SSBs pattern far from the central peak. This choice was based on two factors: (1) the lack of a well-defined crystallographic structure for each of the three sites, which would make assigning dipolar couplings arbitrary, and (2) the expectation that omitting dipolar interactions primarily affects mostly the nearest SSBs and not the whole manifold of SSBs, as would be the case if the quadrupolar interaction were omitted. For illustrative purposes, the depolarization effects on the nearest SSBs due to oversimplified or omitted dipolar interactions are presented in Figure S4 and Table S1. The simulation highlights that the closest SSBs are more affected by the omission/oversimplification of dipolar contributions.

**Fig. 5:**
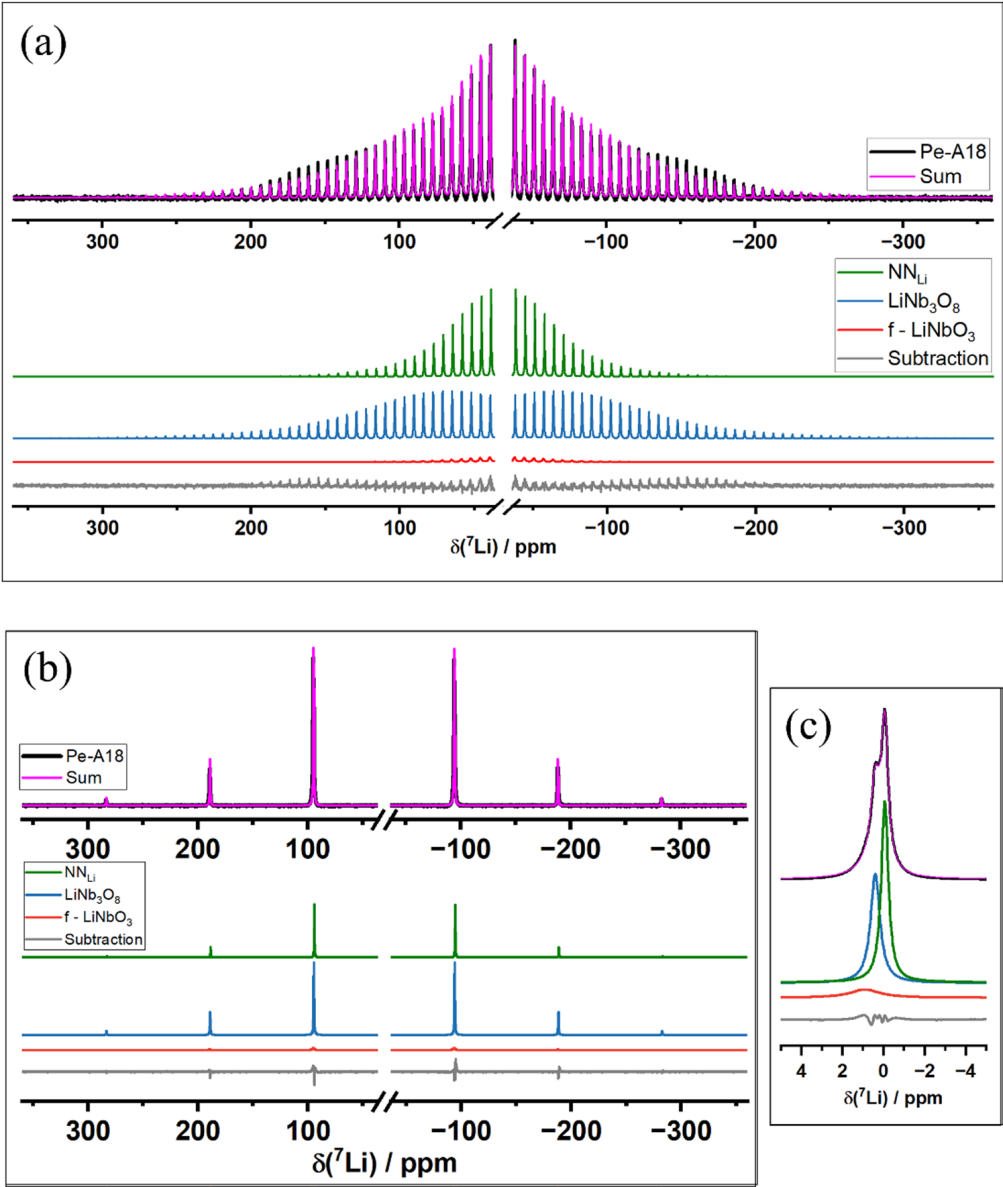
(**a**) From the top to the bottom: ^7^Li spectra of Pe-A18 sample acquired at 2 kHz (black), corresponding simulation (magenta), and individual site contributions—NN_Li_ (red), LiNb_3_O_8_ (green), f-LiNbO_3_ (blue)—followed by the difference between the experimental and simulated spectra (gray). (**b**) Same data as in (**a**) but acquired at 22 kHz. (**c**) Zoomed-in view of the central peak from spectrum (**b**), highlighting the detailed region around the isotropic signal.

Finally, Fig. 5 presents the ^7^Li MAS NMR spectrum and its simulation for Pe-A18 at (a) 2 kHz and (b) 22 kHz, while Fig. 5c zooms the central transition of spectra depicted in Fig. 5b. To account for the local distortion which emerges by growing a single crystal inside a NN matrix, the quadrupolar parameters were calculated using the Czjzek model^34^. At 2 kHz spinning, strong dipolar interactions are insufficiently averaged, leading to SSBs closer to the central transition and complicating accurate simulation. Therefore, the central peak and the first five SSBs and the central peak were excluded from the simulation. In contrast, at 22 kHz, the spinning rate effectively averages dipolar couplings, allowing the full line shape to be simulated. Among the sites, site named LiNb_3_O_8_ reveals the largest ⟨C_Q_⟩, while NN_Li_ and f-LiNbO_3_ have similar values within experimental error. The parameters extracted from the simulation of ^7^Li MAS NMR for Pe-A18 at 2 kHz and 22 kHz are summarized in Table 4.

**Table 4:** Parameters obtained from the spectral simulation of the Pe-A18 sample. For each site, the isotropic chemical shift (δ_iso_), the average quadrupolar coupling constant ⟨C_q_⟩, and the line broadening (lb) are listed above, along with the corresponding area for each site (A).

Samples	Phase	δ_iso_/ppm (± 0.1)	⟨C_q_⟩/kHz (± 10)	A/% (± 4)	L/Hz (± 20)	G/Hz (± 40)
2 kHz	NN_Li_	‒0.3	55	43	132	0
	LiNb_3_O_8_	0.2	106	47	142	0
	f-LiNbO_3_	0.7	53	10	500	0
22 kHz	NN_Li_	‒0.3	55	46	95	0
	LiNb_3_O_8_	0.2	94	45	129	0
	f-LiNbO_3_	0.7	53	9	500	0

Density Functional Theory calculations were performed to (1) compare experimental and theoretical NMR parameters based on the crystallographic structure of LiNbO_3_ and (2) predict the NMR parameters of LiNb_3_O_8_ from first principles. Starting from the crystallographic information file, the atomic positions were optimized to minimize the total energy of the system. Following geometry optimization, the chemical shielding tensors were calculated, and the isotropic chemical shifts were referenced to LiCl, which was used as the standard. The DFT results for C_Q_ and δ_iso_ values for the two crystallographic components investigated are summarized in Table 5. Additional values can be found in Table S2.

**Table 5:** Parameters extracted from DFT calculations. For LiNbO_3_ (8 × 8x4) (ICSD: 84,578) and LiNb_3_O_8_ (8 × 6 × 3) (ICSD: 2921), the isotropic chemical shift (δ_iso_), and quadrupolar coupling constant (⟨C_q_⟩) are reported. ^(a)^Values of NaNbO3 are extracted from literature^35^.

		δ_iso_/ppm	δ_aniso_/ppm	η_cs_	|C_q_|/kHz	η_Q_
LiNbO_3_	^7^Li	1.2	− 0.5	0.4	12	0.1
LiNb_3_O_8_	^7^Li	0.7	2.5	0.5	40	0.6

		δ_iso_/ppm (± 10)	δ_aniso_/ppm (± 5)	η_cs_ (± 0.1)	|C_q_|/MHz (± 1)	η_Q_ (± 0.1)
LiNbO_3_	^93^Nb	− 1004	− 167	0.0	21	0.0
LiNb_3_O_8_	^93^Nb(1)	− 1082	317	0.2	54	0.4
	^93^Nb(2)	− 1102	409	0.2	49	0.5
	^93^Nb(3)	− 1060	426	0.3	67	0.1
NaNbO_3_-Pbcm^(a)^	^93^Nb	− 1078	− 60	0.3	19.5	0.7
NaNbO_3_-P2_1_ma^(a)^	^93^Nb	− 1078	− 90	0.5	20.3	0.7

**Fig. 6:**
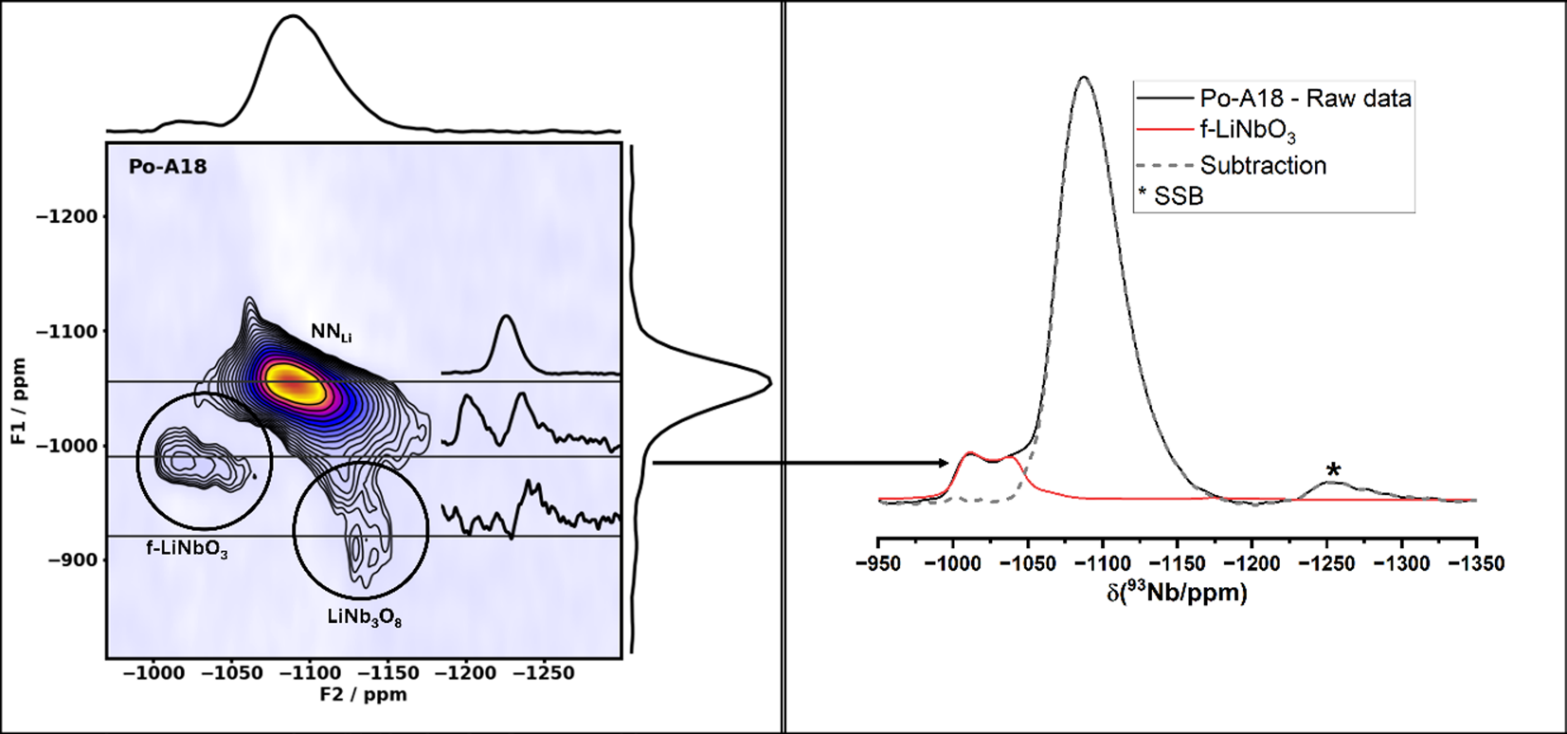
On the right, the ^93^Nb MQMAS-NMR experiment on the Po-A18 sample; on the left, the one-pulse experiment. The peak marked with an * illustrate one 1st order pinning side band on the right side.

^93^Nb spectra are presented in Fig. 6 for comparison with the ^93^Nb DFT results. On the left, ^93^Nb MQMAS spectra and, on the right, ^93^Nb MAS-NMR under 24 kHz, the maximal spinning speed available. Because Po-A18 exhibits the same sites as Pe-A18 and, owing to the larger sample amount available, it provides the clearest distinction between the local ^93^Nb environments. The LiNbO_3_ site is clearly resolved, and the ^93^Nb MQMAS-NMR parameters were extracted, resulting in δ_iso_(f-LiNbO_3_) = − 1002 ppm and P_q_(f-LiNbO_3_) = 23 MHz. The strongest peaks in both spectra correspond to NN_Li_. Parameters corresponding to this peak were extracted from the MQMAS experiment, yielding P_q_(NN_Li_) = 21 MHz and δ_iso_(NN_Li_) = − 1070 ppm. The noisiest site in the MQMAS spectra is attributed to LiNb_3_O_8_. Parameters corresponding to this peak were extracted from the MQMAS experiment, yielding P_q_(LiNb_3_O_8_) = 45 MHz and δ_iso_(LiNb_3_O_8_) = − 1020 ppm.

## Discussion

### Mobility

In stage (a), Figure S1 confirms that fast Li species with correlation times shorter than the millisecond timescale, which would be characterized by a sharp spectral lineshape, were not detected. The slight decrease in T_1_ with increasing temperature observed in the at Fig. 1d suggests that the system is still far from reaching the predicted T_1_ minimum in Bloembergen, Purcell theory^24,36^. As evidenced by Figure S1 (bottom, left), the activation energies calculated from the T_1_ experiments are roughly an order of magnitude smaller than the typical activation energies for Li ion mobility in niobates. This suggests that ion mobility can be excluded as a major contributor to T_1_. Although, the T_1_ relaxation data seem to exhibit some temperature dependence between 400 and 450 K, no significant difference is observed between 300 and 400 K within experimental error. Consequently, using only two points to fit a linear trend—considering the experimental error—would likely introduce substantial uncertainties. Furthermore, no change in the full width at half maximum (FWHM) of the central ^7^Li is observed over a 150 K temperature range. This indicates that the correlation time (τ_c_) remains outside the optimal range for efficient spin–lattice relaxation, implying that the T_1_ minimum is likely shifted to much higher temperatures. Therefore, temperatures as high as 450 K are still considered part of the low-temperature range of spin–lattice relaxation in this material.

In stage (b) in Fig. 1, a phase transition of the LNN18 material occurred at 450 K. Figure S2 depicts the sample holder before stage (a) and after the full cooling down, at the end of stage (b). The clear change in color provides strong evidence of a previously mentioned phase transition, which was so far unreported in this material. Also, Figure S2 (bottom) presents the ^23^Na MAS spectra of central peak of aged LNN18 before heating and immediately after the cooling process. The distinct ^23^Na environments confirm the phase transition in Pe-A18 at 450 K.

For this reason, SED experiments were carried out on a new Pe-A18 sample that had not undergone the phase transition. The experiments were conducted below the transition temperature, 450 K, in order to probe potential slow-lithium species.

Spin–lattice relaxation, magnetization diffusion (spin flip-flop dynamics), and ^7^Li ion mass exchange are the three main factors influencing signal decays in the SED experiment. As illustrated by Figure S3, while the relaxation time *T*_*n*_ is in the order of 0.5 s, the longitudinal relaxation time T_1_ ranges from 5 to 10 s, already suggesting that spin–lattice relaxation is not the dominant contribution. The analysis of the relaxation times *T*_*n*_ as a function of temperature, shown in Fig. 2, confirms a second trend: the plot of inverse temperature versus the logarithm of relaxation times *T*_*n*_ reveals no detectable temperature dependence. This indicates that ion mobility can be excluded as a major contributor to overall decay in SED curves, in which case the data would be fitted with an Arrhenius model. The temperature independence of the decay highlights the predominant role of spin diffusion driven by dipolar interactions. Therefore, we assume that local field fluctuations caused by local lithium-ion motion and vibrational motions in the coordination sphere of the ions are the main contributors to T_1_. These processes, however, do not do not produce motional narrowing of the spectra or affect the stimulated-echo amplitude, which is controlled by spin diffusion.

This finding agrees with the literature on both topics: (a) the size of the crystal and (b) the aging steps. Regarding (a), the size of crystallites, according to the literature^13,25,28^, on the order of tens of nanometers exhibit a high interface area to volume ratio. Since interfaces tend to show more defects, such as oxygen vacancies, the ionic mass exchange of lithium (or oxygen vacancies) is also more likely to occur at the interface of the crystalline material. In Xia et al.^13^, the tungsten atom substitutes on a niobium site, which produces a lithium vacancy in the lithium sublattice. This replacement creates a mechanism to induce mobility in lithium. In contrast, for crystallites in the order of hundreds of nanometers, the reduced interface-to-volume ratio significantly limits the presence of interface defects and, consequently, decreases the ionic mass exchange of lithium. Additionally, (b) the aging steps also play a role in decreasing the oxygen vacancies present inside the material. The study made by Yu et al.^12^ illustrates this effect very clearly. Due to aging under atmospheric conditions, the O_2_ gas present oxidizes the material, mediating the decrease in oxygen vacancies within the structure^11,25,26,37^. This finding reinforces that the any peaks observed in the ^7^Li NMR spectrum might be attributed to local ^7^Li structural environments.

### Structural investigation

Contrast variations observed in TEM images within the LiNbO_3_ matrix is possibly arising from distinct structural ordering. Among these features, lamellar structures characterized by alternating light and dark bands were identified, as illustrated in Fig. 3c, indicating the presence of organized nanoscale domains. In ceramic materials, such lamellae can also be associated with phase separation, chemical composition variations, or structural defects generated during thermal processing. In the present case, these observed features may reflect nanoscale phase separation between LiNbO_3_ and LiNb_3_O_8_ or Li_3_NbO_4_, revealing the formation of Li-poor and Li-rich phases. The first phenomenon could result from lithium evaporation during high-temperature synthesis exposed to an O_2_-rich atmosphere. NMR was, then, employed to probe the local environment of lithium atoms and lithium distribution beyond TEM resolution.

Since broadening caused by motion can be excluded from this interpretation, the analysis of the central peaks is based on the distribution of isotropic chemical shift values but, more importantly, on the broadening caused by dipolar interactions. This is illustrated in Figure S5 with ^6^Li(^7^Li) CPMAS spectra obtained from samples with natural abundance of ^6^Li. The ^6^Li(^7^Li) CPMAS experiment demonstrates that the distribution of distinct chemical environments is very narrow, as evidenced by the sharp ^6^Li lineshape. In contrast, the central peak in ^7^Li MAS spectra is much broader than in ^6^Li, confirming that the broadening is dominated by residual dipolar interactions, which are not fully averaged under MAS. This demonstrates that the broadening in ^7^Li originates primarily from dipolar coupling rather than from chemical shift heterogeneity.

The broader line shape observed in Pe-U18 in comparison with Po-U18 sample (Fig. 4) was interpreted based on differences in the quenching process—also interpreted in the basis of dipolar interaction. While it may seem counterintuitive that quenching reduces spectral broadening, this can be rationalized: generally speaking, quenching increases compositional disorder and broadens chemical shifts. In perovskite alkaline niobates, quenching LNN18 at 1300 °C—temperature which LiNbO_3_ is soluble in the NaNbO_3_ matrix—enhances the compositional disorder of ^23^Na through variations in quadrupolar couplings^38^. For ^7^Li, although quenching would be expected to increase the distribution of chemical environments, this effect is compensated by the reduction of dipolar broadening as the Li–Li distances increase. As a result, the overall outcome favors a narrowing of the ^7^Li line, since quenching suppresses the formation of LiNbO_3_, and the line narrowing outweighs the expected broadening from chemical shift distribution.

The unaged Po-U18 sample behaves as expected, with only the NN_Li_ site present, corresponding to lithium fully dissolved in the sodium–niobium matrix. In contrast, Pe-U18 sample exhibits an additional broadened shoulder at higher frequencies (Site i-LiNbO_3_, Fig. 4a). Chemical shifts resonating at higher frequencies may indicate shorter Li–O bond lengths, resulting from lattice contraction in an environment with smaller sodium concentration. Consequently, higher isotropic chemical shifts are expected due to an enhanced degree of bond covalency, as well established in the literature^39,40^.

Broadened shoulder can be rationalized by the thermal gradient that occurred under quenching. During the quenching process, it is well known that the dimensions of a sample have an influence on the thermal gradient^41^ and hence the chemical homogeneity obtained from the sample. Specifically, transient temperature gradients are higher with thicker samples^41^. Based on this, we hypothesize that the quenching process may be more effectively achieved in the powdered sample. In contrast, the smaller interface area of the pellets (10 mm vs. 1.5 mm) would reduce heat exchange with the ambient air during quenching, allowing molecular dynamics to persist over a longer cooling period. Study on relaxor transition temperatures in Na_1/2_Bi_1/2_TiO_3_–BaTiO_3_ pellets of varying thickness^42^ shows that air quenching—the sample is removed from the oven and exposed to ambient air—from 1100 to 425 °C can take up to 100 s, enough for nanoscale rearrangements on a length scale of few Ångströms observable by NMR but undetectable in macroscopic measurements. In Pe-U18, these nanoscale rearrangements that might be induced by the thermal gradient during quenching may lead to an incipient formation of LiNbO_3_, facilitating magnetization diffusion, broadening the lineshape as observed in Site i-LiNbO_3_. Consequently, local environments with higher lithium concentrations than expected in a homogeneous quenched matrix may form.

The spectral simulation of the aged samples shows three peaks: (a) Site NN_Li_, (b) Site f-LiNbO_3_ and (c) Site LiNb_3_O_8_. The peak labeled as Site NN_Li_ is again attributed to lithium in the NN matrix, as it exhibits lineshape parameters similar to those of the corresponding unaged samples (Table 3). This strong similarity indicates that these lithium ions remain within the sodium–niobate matrix after aging. Site f-LiNbO_3_, by its turn, was associated with the formation of a secondary phase, namely LiNbO_3_ precipitates, as evidenced by its significant broadening—attributed to, mainly, enhanced dipolar interactions, as discussed in Figure S5. This assignment is further supported by ^93^Nb MAS and MQMAS NMR, discussed above, and by previous X-ray scattering studies on these samples by Rödel and coworkers^18,22^. Regarding Site LiNb_3_O_8_, the sharper lineshape shows a similar linewidth to that of NN_Li_, suggesting that site labelled as LiNb_3_O_8_ is also lithium-poor site. Studies on thin films have indicated that LiNb_3_O_8_ forms mainly after longer annealing times, especially under an O_2_ atmosphere, due to Li_2_O evaporation^43,44^. During ceramic thermal treatment, the driving force for crystal growth tends to incorporate lithium into the forming crystal, while the surrounding regions may become lithium-deficient, promoting the formation of the LiNb_3_O_8_ phase, which lies on the Nb-rich side of the Li_2_O–Nb_2_O_5_ phase diagram near the composition of LiNbO_3_^45^. Based on these considerations, LiNb_3_O_8_ was initially hypothesized as the label of this site.

Further investigation was performed to verify whether this site truly corresponds to LiNb_3_O_8_, combining the analysis of quadrupolar interaction analysis by both NMR and DFT calculations. As this analysis aims to provide insight into a possible correlation between this third site observed by NMR in the aged samples and the lamellar structure revealed by TEM in Pe-A18, only the Pe-A18 sample was selected for this detailed study.

The ^93^Nb NMR results (Fig. 6) provide consistent evidence for the presence of LiNb_3_O_8_. Three distinct sites are observed in the ^93^Nb NMR spectra: (a) niobium in sodium–niobium NN, with NMR parameters in very good agreement with the DFT parameters reported by Johnston et al. (b) LiNbO_3_, with NMR parameters in excellent agreement with DFT calculations; and (c) the third site with NMR parameters consistent with those expected by DFT calculation for LiNb_3_O_8_. The low signal-to-noise ratio observed for this latter site is expected for the following reasons. The MQMAS spectra reveal a better triple- to single-quantum conversion at lower quadrupolar coupling constants than those expected for LiNb_3_O_8_, compromising the detection of this site^46^. In addition, the spinning frequency limitation of only 24 kHz for ^93^Nb measurements reduces the possibility of observing distinct sites in a one-pulse experiment, due to the overlap of very closely spaced spinning sidebands with the broad anisotropic distribution.

The analysis of quadrupolar interaction for ^7^Li NMR (Fig. 5) also reinforce the presence of LiNb_3_O_8_. The previous three distinct sites—(a) NN_Li_, (b) LiNbO_3_ and (c) LiNb_3_O_8_—were used to simulate the whole ^7^Li NMR under distinct MAS rates spectra. In the literature^33^, the values for ^7^Li NMR quadrupolar parameters of LiNbO_3_ calculated by first-principles range from 8 to 37 kHz, whereas experimental values are found in the range of 53 to 54 kHz^33,47,48^. In the current work, the value found in DFT calculations for LiNbO_3_ is 12 kHz and the experimental value of pure LiNbO_3_ (99.9% Sigma Aldrich) exhibit values among 36 kHz and 48 kHz, as illustrated by Figure S6. Both, in the literature and in the present study, the DFT results show a small discrepancy with respect to NMR measurements. We believe that discrepancies might be attributed to distortions, defects, or Li vacancies present in real samples, which are certainly not represented in the idealized DFT structure. For the LiNb_3_O_8_ site, NMR observations are in agreement with DFT predictions, both indicating a stronger quadrupolar interaction than expected for LiNbO_3_, along with chemical shifts shifted toward more negative values. However, the ⟨C_Q_⟩ value obtained from MQMAS NMR lineshapes for LiNb_3_O_8_ is smaller than the value predicted by DFT calculations. Such a deviation can be again attributed to the reduced MQMAS efficiency at large quadrupolar couplings, which compromises the detection of this site.

**Fig. 7:**
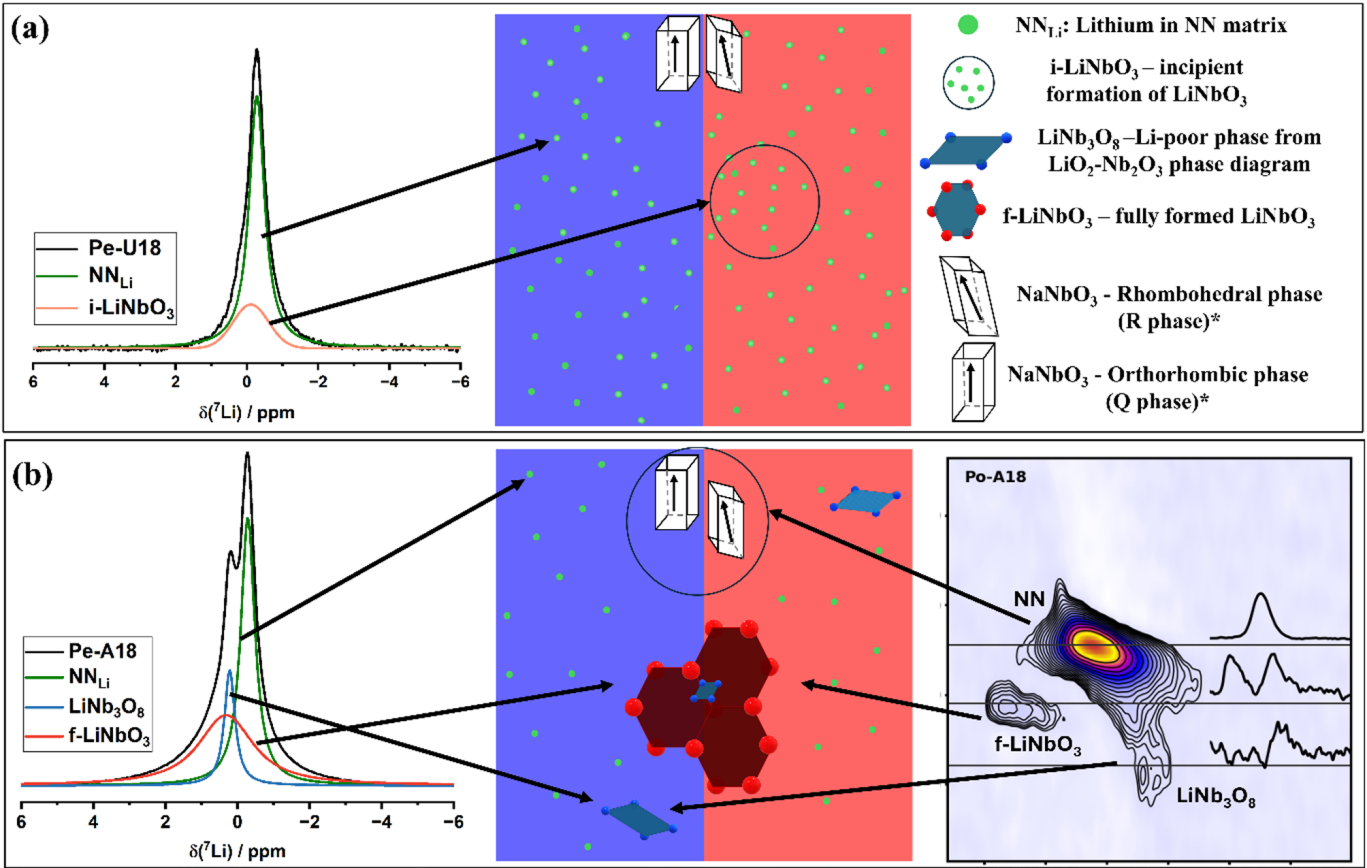
Illustration of proposed hypotheses for short range order in (**a**) Pe-U18 sample, and (**b**) Pe-A18. The * mark in both NaNbO_3_ are information about sodium short-range order extracted from the literature^18,38^.

Finally, the combined DFT and NMR results on ^93^Nb and ^7^Li NMR support the assignment of LiNb_3_O_8_ as a plausible candidate the additional peak detected in the NMR spectra, which can correspond to the lamellar structures observed in TEM images. Figure 7 presents a schematic interpretation of the ^7^Li and ^93^Nb NMR signals observed in the pellet samples.

Summarizing the above, Fig. 7 provides an overview of the discussion on the^7^Li and ^93^Nb MAS NMR spectra. In Fig. 7a, Site NN_Li_ represents lithium homogeneously distributed within the sodium–niobium matrix. Its fingerprint is the sharp lineshape, resulting from reduced dipolar interactions caused by quenching the sample when Li is dissolved in the sodium–niobium framework. The unaged Pe-U18 sample also appears to contain incipient LiNbO_3_. Nanoscale rearrangements induced by the thermal gradient during quenching in pellets may promote the initial formation of LiNbO_3_. As suggested by NMR results, the site i-LiNbO_3_ resembles NMR parameters of those of LiNbO_3_ but with weaker dipolar interactions. This incipient ordering facilitates magnetization diffusion, leading to broader lineshapes, as observed for Site i-LiNbO₃ in comparison with NN_Li_. Higher chemical shift values are also detected, which are characteristic of shorter Li–O bond lengths, a consequence of local lattice contraction in regions with lower sodium concentration relative to NN_Li_. Figure 7b illustrates the lattice after the formation of precipitates, along with the corresponding ^7^Li MAS NMR central peak. Again, lithium homogeneously distributed within the sodium–niobium matrix is represented by NN_Li_ site. Also, the formation of LiNbO_3_ is supported by ^93^Nb and ^7^Li DFT and NMR simulations and labeled as f-LiNbO_3_. A third site, not detected in previous XRD studies on the same samples, is indicated by TEM results and investigated by ^7^Li and ^93^Nb NMR. This site exhibits a stronger quadrupolar interaction for both ^93^Nb and ^7^Li compared to the other two sites, along with a weaker dipolar interaction. It was tentatively assigned to LiNb_3_O_8_. During aging in air, LiO_2_ evaporation may occur, favoring the formation of LiNb_3_O_8_, a Nb-rich phase within the Li_2_O–Nb_2_O_5_ phase diagram, close to the composition of LiNbO_3_.

## Conclusion

In conclusion, NMR analysis of magnetization dynamics indicates that spin–echo decay is dominated by spin diffusion and dipolar interactions, rather than by ⁷Li ionic exchange. These findings align with previous reports highlighting the impact of crystallite size and thermal treatment on lithium mobility and oxygen vacancy concentrations. Furthermore, solid-state ⁷Li and ^93^Nb NMR was employed to probe lithium dynamics and the local structure in aged LNN18, revealing pronounced effects of thermal processing on lithium ordering. Variations in dipolar interaction parameters indicate that quenching suppresses the formation of lithium-rich phases, while aging promotes the controlled growth of LiNbO_3_ precipitates. The ⁷Li spectra of aged LNN18 samples reveal multiple lithium environments: (i) Li homogeneously distributed in the sodium–niobium matrix (NN_Li_), (ii) Li in LiNbO_3_ (f-LiNbO_3_), and (iii) a third environment, tentatively assigned to LiNb_3_O_8_. The latter assignment is supported by its dipolar interaction, isotropic chemical shifts, and quadrupolar coupling investigations made by DFT and NMR simulations. This third phase is also consistent with the lamellar features observed in TEM images, which likely arise from local contrast between Li-rich and Li-poor regions. While TEM provides morphological evidence, solid-state NMR enables quantification of the relative populations of each lithium site, offering deeper insight into this additional environment and rationalizing the TEM observations. Altogether, this work advances the understanding of local ⁷Li environments in LNN ceramics and their evolution upon thermal aging.

## Material and methods

### Material

The first batch of samples was fabricated using the powders of Na_2_CO_3_ (99.5%, Alfa Aesar), Li_2_CO_3_ (99.0%, Alfa Aesar), and Nb_2_O_5_ (99.9%, Alfa Aesar). The raw materials were ball-milled before being calcined at 850 °C. The calcined powders were cold-pressed isostatically compacted into pellets of dimensions of 10 mm × 1.5 mm at a pressure of 357 MPa. The pellets were then sintered at 1300 °C for 2 h, heat treated at 1300 °C for another 2 h followed by air quenching, i.e., rapidly cooled from 1300 °C to room temperature in air bath. The air quenching step was chosen since previous studies have shown that this method does not typically induce cracks, with micro-cracking occurring only under more severe conditions such as liquid-nitrogen quenching^42,49^. Moreover, Takagi et al*.* demonstrated that controlling the cooling gradient with an air fan can enhance the mechanical strength of quenched BNT samples, and that even at the highest air-quenching rates no undesired cracks were formed^50^. The unaged pellet with composition 18 LiNbO_3_–82 NaNbO_3_ (or LNN18 sample) was named Pe-U18. In order to promote the Li-rich phase, the sample was aged in two-step thermal treatment: the first at 500 °C for 24 h, followed by a second aging step at 600 °C for 8 h. This aged LNN18 sample was named Pe-A18. More details about the sample fabrication can be found in Zhao et al.^18^ Samples of the same batch and identical thermal history were used in TEM images.

Due to the presence of thermal gradients during the quenching of a 10 mm × 1.5 mm pellet, a second batch of samples was prepared as a reference to investigate the quenching effects. For this batch, high-purity raw materials—Na_2_CO_3_ (99.999%, Aladdin Industrial Corporation Co. Ltd.), Li_2_CO_3_ (99.99%, Aladdin Industrial Corporation Co. Ltd.), and Nb_2_O_5_ (99.99%, Aladdin Industrial Corporation Co. Ltd.)—were used. Unlike the first batch, the calcined powders were not cold-pressed into pellets but were instead left in their powdered form, thereby creating a larger surface area that allowed more efficient thermal exchange with the ambient air and more homogeneous cooling during the quenching. Aside from this variation, all other processing steps were identical to those previously described. The resulting quenched, unaged powder sample with LNN18 composition was designated as Po-U18. To promote the formation of a Li-rich phase—such as LiNbO_3_ (LN)—the sample underwent the same two-step aging treatment: first at 500 °C for 24 h, followed by 600 °C for 8 h. The resulting aged powder sample was named Po-A18. Further details on the fabrication process can be found in Li et al.^51^.

### Solid-state techniques and setup

#### Line-shape analysis

The ^7^Li NMR line shape is primarily influenced by first-order quadrupolar interactions^24,27,48^. As a result, the central transition is not broadened by quadrupolar interaction and is instead primarily governed by dipolar interactions. At lower-field (approximately ≤ 4 T), the linewidth is broadened by the dipolar interaction and the spectra will resonate at Larmor frequency—often with no indication of site differentiation. At higher fields (approximately ≥ 7 T) and with the application of Magic Angle Spinning technique^52^, which will be further explained below, the chemical shift interaction dominates the resonance frequency of the central peak—enabling a potential resolution of distinct lithium sites. Moreover, all the interactions mentioned above—chemical shift, quadrupolar, and dipolar—are anisotropic, leading to spectral broadening. As mentioned, depending on the magnetic field strength and/or the technique used, a particular interaction may dominate the spectrum and can be investigated more accurately.

For the elucidation of local sites with small differences in NMR parameters, these broadenings complicate the investigation of local structures, as many distinct lineshapes are obscured by anisotropic interactions, making it difficult to resolve individual sites. One way to overcome this obstacle is using MAS technique^52^. By spinning the sample at the magic angle of 54.7° (the zero of the first Legendre polynomial), the ^7^Li lineshape first-order anisotropic interactions will be averaged out, resulting in a central peak with SSBs spaced by the rotation frequency and modulated by the anisotropic interaction. Using MAS technique, high resolution is achieved for the investigation of local structures or sites hidden by anisotropic interactions^52–54^.

Complementarily, the analysis of static ^7^Li spectrum can bring valuable contributions to the internal mobility of the system. The ^7^Li line-shape comprises two components: the central transition, $\left|1/2\right.\rangle \to \left|-1/2\right.\rangle$, which displays a narrower line, and the satellite transitions, $\left|3/2\right.\rangle \to \left|1/2\right.\rangle$, $\left|-1/2\right.\rangle \to \left|-3/2\right.\rangle$, which produce a broader line. While the central transition is unaffected by the quadrupolar interaction and is predominantly influenced by dipolar interaction, the satellite transitions are broadened by the anisotropy of quadrupolar interaction^24,27,55^.

The spectrum can also be modified by the presence of mobile lithium species if the ionic jumps happen with jump rates on the order of the strength of the quadrupolar interaction or faster, which cause motional induced line-shape changes, e.g., the narrowing of the spectrum^11–13^. Typically, for ^7^Li, the quadrupolar interaction, represented by the quadrupolar coupling constant, is in the order of 1 to 10^2^ kHz, therefore the lineshape change will be sensitive to inverse jump rates on the order of 10 µs to ms or faster. Jumping frequencies considerably smaller than this are ineffective on the NMR time scale and will, therefore, not change the spectral line-shape.

#### Two-time correlation function

The SED technique, can be used to study slower jumping processes with inverse rates in the millisecond to second range^27^. The SED sequence is 90°_y_ − *t*_*e*_ − 45°_x_ − *t*_*m*_ − 45°_x_ − *t*_*e*_, where the evolution time t_e_ should be much smaller than the average ^7^Li ion jump time, and t_m_ is varied and on the order of the average jump time. Hence, during tₑ, the frequency is labeled, and during *t*_*m*_, the dynamics may occur. The signal decay in this pulse sequence is related to three factors: spin–lattice relaxation, magnetization diffusion (spin flip-flop movement), or ionic mass exchange. Since magnetization diffusion occurs through dipolar interaction, it is roughly independent of temperature. Therefore, temperature variation separates the contributions of ionic mobility and magnetization diffusion. Finally, the decay of stimulated echo experiment can be fitted to a stretched exponential function (or KWW function)^24,27,55^:

1 $$S({t}_{m}, {t}_{e})\propto exp \left[- {\left(\frac{{t}_{m}}{{T}_{n}}\right)}^{\beta }\right]$$

where 0 < *β* < 1 and T_n_ is a relaxation time which quantifies the time of full magnetization loss.

The stretching parameter *β* can be interpreted as an indicator of an ordered structure when *β* → 1, which corresponds to a mono-exponential decay. For *β* < 1, multiple distinct time scales are involved in the decay, meaning that the smaller *β* is, the more pronounced the dispersion in relaxation times.

#### Setup

##### Two NMR setups were employed for the ^7^Li NMR measurements

(a) For high-resolution, a 3.2 mm MAS probe was used in a Bruker AVANCE III 600 MHz spectrometer, in which ^7^Li and ^93^Nb show a Larmor frequency of, respectively, 233.23 MHz and 146.88 MHz. Spectra simulations were performed using ssNake and Simpson^56,57^. To quantify the SSBs, a flip angle of 90° was used to improve the signal to noise ratio.

All the ^7^Li measurements were performed using a nutation frequency corresponding to 72 kHz for 0.1 M LiCl. For ^7^Li, single pulse experiments were performed under distinct spinning rates from 2 kHz up to 22 kHz. Single pulse experiments used to quantify the sites of the spectra were measured under a flip angle of 30° and a relaxation delay corresponding to 5·*T*_1_ = 150 s.

^6^Li(^7^Li) Cross Polarization MAS experiments were performed on a Bruker Avance Neo operating at a magnetic field of 9.4 T, using a 4.0 mm solid-state probe and heteronuclear ^7^Li decoupling with the SPINAL technique. The spinning frequency of 7.0 kHz and contact time of 8500 ms was used. Relaxation delay was set to 3∙T1 corresponding to 57 s for Pe-U18 and 400 s for LiNbO_3_. The chemical shift was referenced to 0.1 M LiCl.

All the ^93^Nb measurements were performed using a nutation frequency corresponding to 68 kHz for NbCl_5_ saturated in acetonitrile solution. For ^93^Nb, single pulse and MQMAS experiments were performed under 24 kHz. Single pulse experiment used to verify the sites of the spectra were measured under a flip angle of 22.5° and a relaxation delay corresponding to 5·*T*_1_ = 2 s. MQMAS experiment were performed under excitation and conversion MQMAS pulse of 2.35 and 0.78, respectively and a third soft pulse corresponding a 90° flip angle of 18.5 μs. MQMAS experiment was optimized directly on the sample.

(b) A home-made probe was used for measuring ^7^Li in variable-temperature experiments over the range of 300 K to 450 K. In this setup, a superconducting magnet operating at a ^7^Li Larmor frequency of 62.9 MHz was employed. Using this system, the ^7^Li spin lattice relaxation curves were obtained by saturation recovery and fitted with Eq. (2). ^7^Li static spectra were measured using the solid-echo sequence, 90° − *τ* − 64° − *τ*, with *τ* = 20 μs delay^24,27,37^. The SED sequence, 90° − *t*_*e*_ − 45° − *t*_*m*_ − 90° − *t*_*e*_, was used for an evolution time of *t*_*e*_ = 20 μs. The results were fitted with Eq. 1. In neither of the two pulse sequences, the 90° pulse lengths exceeded 2.5 μs. The temperature was controlled within ± 0.5 K by liquid nitrogen cryostat in the superconducting magnets.

2 $$S\left(t\right)=S(0)\left\{1-exp \left[- {\left(\frac{t}{{T}_{1}}\right)}^{\beta }\right]\right\}$$

### TEM

Bright-field and dark-field imaging, selected area electron diffraction pattern, scanning transmission electron microscopy, and element mapping were conducted using a JEOL JEM-2100F microscope (JEOL, Tokyo, Japan), fitted with an energy-dispersive X-ray spectroscopy detector (X-Max80, Oxford In­struments, Abingdon, UK). High-resolution STEM analyses were performed on a JEOL JEM-ARM 200F microscope featuring spherical aberration correction. For TEM sample preparation, ceramic specimens—both before and after aging—were sectioned into discs of 3 mm diameter and 300 μm thickness. These discs were mechanically thinned to approximately 20 μm through sequential polishing steps. To alleviate residual stress from mechanical preparation, the foils were thermally annealed at 200 °C for 0.5 h with controlled heating and cooling rates of 1 °C/min. The as-annealed thin foils were subsequently mounted onto molybdenum grids and further ion-milled (Gatan model 600, USA) until reaching electron transparency. Each aging condition corresponded to a separately prepared TEM specimen, randomly extracted from the bulk ceramic discs. Electron transparency areas generally encompassed multiple grains with various orientations, allowing the examination of precipitates to be distributed relatively uniformly throughout the grain interior [22]. Detailed microstructural characterizations were concentrated on selected LiNbO_3_ precipitates and their surrounding matrix regions. The aspect ratio was determined statistically by measuring the length and the width of 100 edge-on precipitates imaged along the [100]_PC_ direction.

### DFT

First-principles DFT calculations were performed using the CASTEP code^58^ to investigate the structure and NMR parameters of the crystals (1) Li_3_NbO_4_ (ICSD 243,922)^59^, (2) LiNbO_3_ (ICSD 84,578)^60^, and (3) LiNb_3_O_8_ (ICSD 2921)^61^. All CIF files for all three structures were indexed with the Inorganic Crystal Structure Database (ICSD, FIZ Karlsruhe) database. All calculations were conducted under periodic boundary conditions. The computational procedure consisted of two stages: (1) Geometry Optimization, followed by (2) DFT-NMR calculations. For both cases, the Perdew–Burke–Ernzerhof revised for solids (PBEsol) exchange–correlation functional within the generalized gradient approximation (GGA) was employed^62^. A plane-wave basis set with a kinetic energy cutoff of 800 eV was used. The Brillouin zone was sampled using a k–point grids of 6 × 6 × 6 (Li_3_NbO_4_), 8 × 8 × 4 (LiNbO_3_), and 8 × 6 × 3 (LiNb_3_O_8_), 10 × 10 × 10 (MgO), and 10 × 10 × 10 (LiCl)^63^. For the geometry optimization, the Broyden–Fletcher–Goldfarb–Shanno (BFGS) algorithm^64^ was employed, with convergence threshold set to 1 × 10^–6^ eV per atom for total energy, 0.02 eV/Å for forces, and 1 × 10^–6^ eV for electronic energy. A smearing width of 0.1 eV was applied to aid electronic convergence.

Subsequently, NMR parameters were calculated using the gauge including projector augmented wave (GIPAW) method^65,66^ as implemented in CASTEP. The magnetic shielding and electric field gradient tensors were calculated^67^. The magnetic shielding tensor components (σ_iso_, σ_iso_, η) were converted to chemical shifts using the relations: δ_iso_ = σ_reference_–σ_iso_; δ_aniso_ = σ_aniso_. For the reference shielding, σ_reference_, the secondary standards, LiCl for ^7^Li (0 ppm) and MgO (47 ppm) for ^17^O, were used. For ^93^Nb, LiNbO_3_ (1004 ppm) was used as an internal reference and Li_3_NbO_4_ and LiNb_3_O_8_ were quoted with respect to it.

The computational parameters were carefully chosen to ensure reliable convergence and accurate prediction of both the optimized crystal structure and NMR properties. The obtained NMR parameters from equivalent sites were averaged and the corresponding standard deviations were calculated.

## Supplementary Information

Below is the link to the electronic supplementary material.

Supplementary Material 1

## Data Availability

Raw data that support the findings of this study are available from the corresponding authors upon reasonable request.

## Abbreviations

LNN: Lithium sodium niobate
DFT: Density functional theory
NMR: Nuclear magnetic resonance
TEM: Transmission electron microscopy
MAS: Magic angle spinning
STEM: Scanning transmission electron microscopy
SED: Stimulated echo decay
