# Supplementary material for: ⁷Li NMR short-range ordering in hardened lithium sodium niobate
Source: Sci Rep. 2025 Oct 9;15:35233. doi: 10.1038/s41598-025-21740-w (PMC12511322; doi:10.1038/s41598-025-21740-w)
Supplement: Supplementary file 1 — Supplementary Material 1 [file 41598_2025_21740_MOESM1_ESM.pdf]

^ # ^{7}Li NMR Short-Range Ordering in Hardened Lithium Sodium Niobate

Millena Lograd[1], Anuraag Gaddam[2], Fangping Zhuo[3], Changhao Zhao[4], Shuang Gao[5], Hergen Breitzke[1], Mohammad Ali Badragheh[6], Markus Rosenstihl[6], Michael Vogel[6], Jürgel Rödel[2], Gerd Buntkowsky[1]

[1] Eduard-Zintl Institute for Inorganic and Physical Chemistry, Department of Chemistry, Technical University of Darmstadt, Darmstadt, Germany

[2] CICECO – Aveiro Institute of Materials, Department of Chemistry, University of Aveiro, Aveiro, Portugal

[3] Division of Nonmetallic-Inorganic Materials, Department of Materials and Earth Sciences, Technical University of Darmstadt, Darmstadt, Germany

[4] State Key Laboratory of Electrical Insulation and Power Equipment, School of Electrical Engineering, Xi'an Jiaotong University, Xi'an, Shaanxi, PR China

[5] Key Laboratory of Green Extraction & Efficient Utilization of Light Rare-Earth Resources (Inner Mongolia University of Science and Technology), Ministry of Education, School of Rare Earth Industry, Baotou 014010, P.R. China.

[6] Institut für Physik Kondensierter Materie, Technische Universität Darmstadt, Hochschulstr. 6, 64289 Darmstadt, Germany

Results

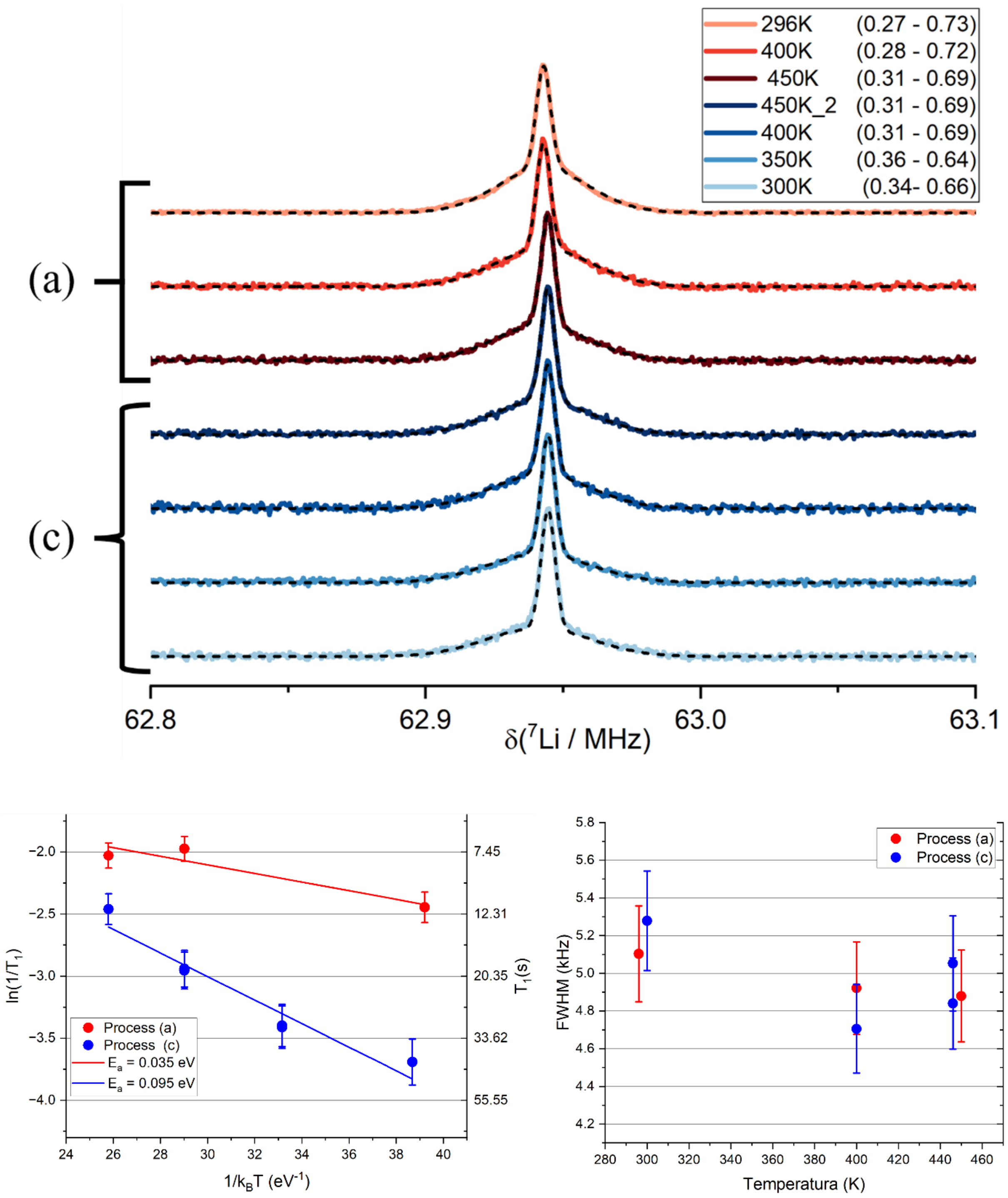

Figure S1: On the top: Static spectra of the ^{7}Li in the processes (a) and (c) of Figure 2. Each line is simulated with two gaussian components, one for central transition (more intense peak) and a second one for both satellite transitions (broader peak). The legend of this graph shows the temperature corresponding to each spectrum and, in parentheses, the areas of the satellite and central transitions, respectively. No significative change is observed in the proportion of the area. On the bottom, left: Supplementary activation energy (E_a) calculation extracted from T_1 relaxation via Bloembergen–Purcell–Pound theory in the low-temperature regime using data of Figure 1 (e) process (a) – which represents the structure before the phase transition. On the bottom, right: supplementary analysis of Full

Width Half Maximum (FWHM) from Figure S1 process (a). No FWHM difference is found over a range of 150 K variation of temperature in the central peak of ^{7}Li.

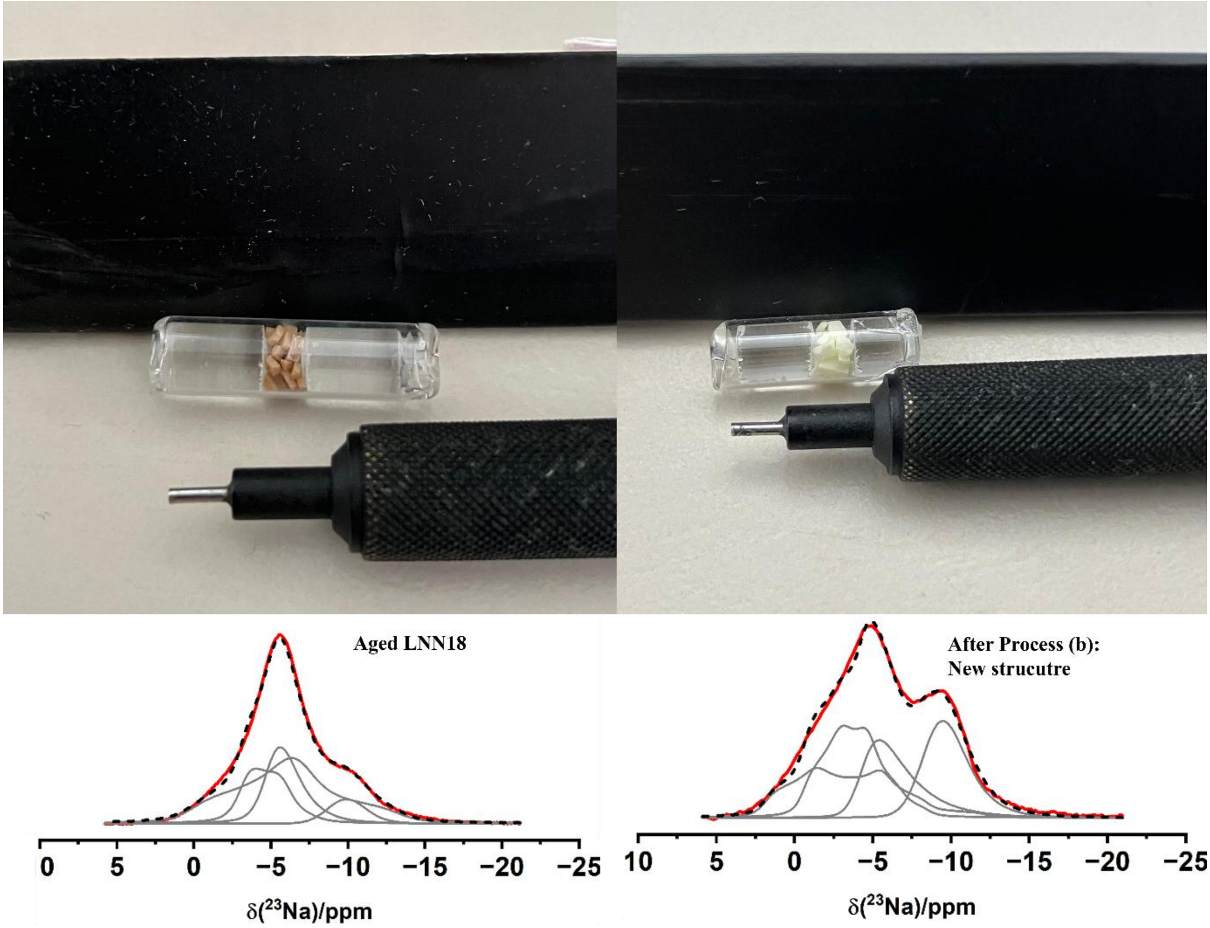

Figure S2: On the left, top: LNN18 inside the sample holder before stage (a) and, on the right, top: sample after the full cooling down illustrated in stage (b) of Figure 1. The color change indicates a phase transition occurring at 450K, as demonstrated by the Temperature vs. T_1 curve. On the bottom, left: ^{23}Na MAS spectra of central peak measured at 10 KHz of aged LNN18 at 10KHz before stage (a) of Figure 2 and, bottom, right, ^{23}Na MAS spectra of aged LNN18 at 10KHz immediately after colling down after stage (b) in Figure 2. The change in the sodium environment emphasizes the phase transition at 450K.

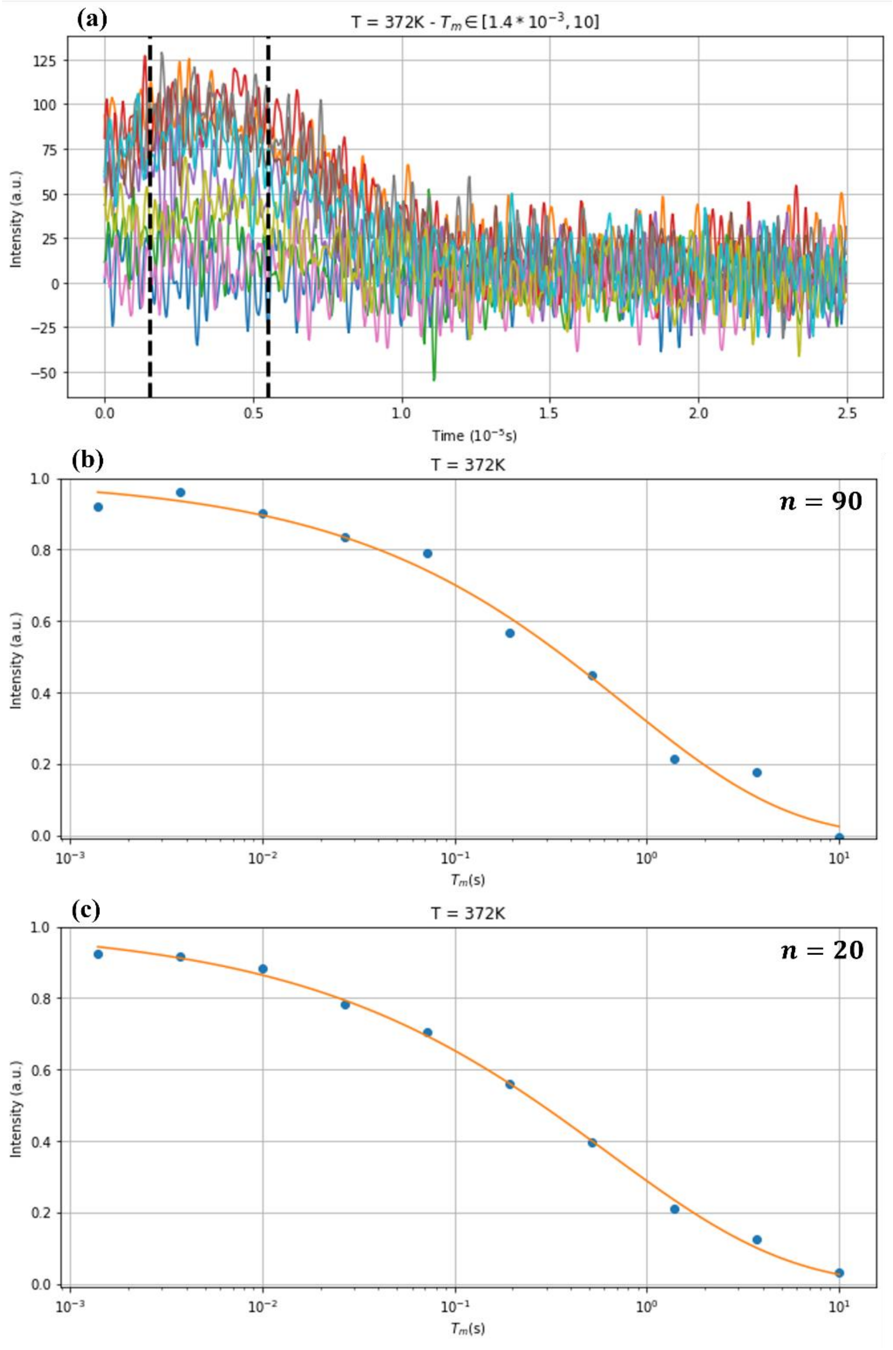

Figure S3: (a) ^{7}Li NMR stimulated-echo decays of a sine-sine correlation function for Pe-A18 at 372 K. Distinct decays result from different T_m values in the range $[1.4\cdot10^{-3},10]$ s. A distinct integration range of [-*n* , *n*] around the maximum was used to estimate the error due to noisy decays. Graphs (b) and (c) show the simulated intensity decay of the sine-sine correlation function for integration between [-90,90] and [-20,20] points around the theoretical maximal peak. Equation (1) was used, and parameters β and T_n were simulated.

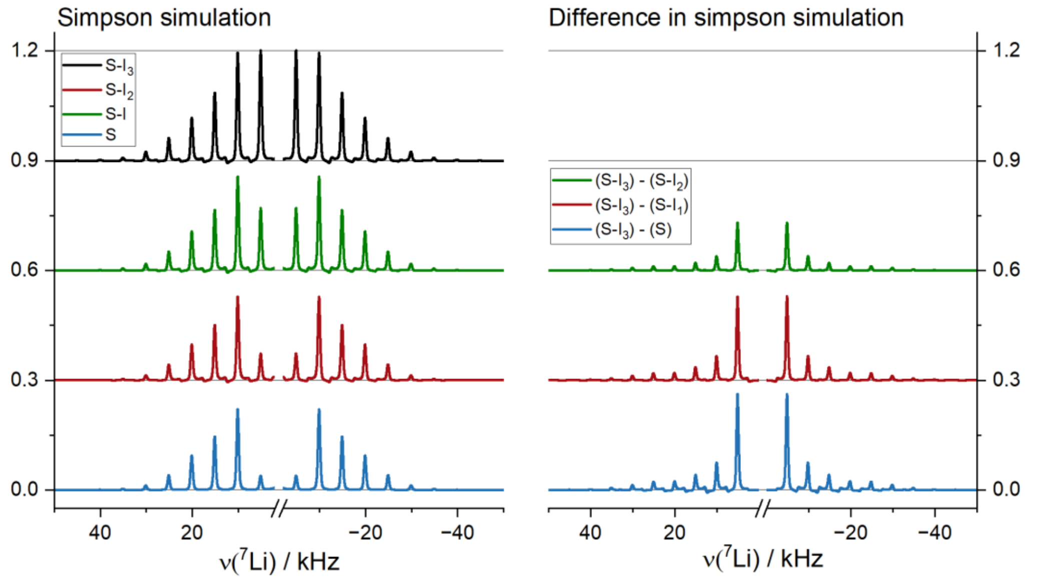

Figure S4: On the right, Simpson simulation at 5kHz and C_q = 91.8 kHz. From bottom to the top, the dipolar coupling constant was chosen as zero and considering only pairwise S–I dipolar interactions (i.e., no S–I–S couplings), the dipolar coupling constant was set to 1281 Hz for two-spin (S–I), 1814 Hz for three-spin ($S–I_1 + S–I_2$), and 2222 Hz for four-spin ($S–I_1 + S–I_2 + S–I_3$) systems — all leading to M_2 values of 65×10^6 $rad^2\cdot Hz^2$. More parameters are described in Tabel S1. On the left, the difference in intensities relative to the four-spin system is illustrated. It is easy to observe that the outer spinning sideband orders are only weakly affected by the simplification of dipolar interactions, whereas in very simple cases, such as the absence of dipolar coupling, the first- and second order spinning sidebands are severely affected. The results illustrate that the SSBs closest to the isotropic peak are the most affected by the simplification or omission of dipolar interactions. More information about the simulation can be found in Table S1.

Table S1: Further SIMPSON parameters used for the simulation of the lineshapes are provided. The Euler angles were set to zero in all $S–I_n$ systems.

	$S-I_3$	$S-I_1$	S-I	S
Dipolar coupling constant	-2222<br>-2222<br>-2222	-1814<br>-1814	-1218	0
Number of points	16384	16384	16384	16384
Spectral windows	630000	630000	630000	630000
Crystal file	rep320	rep320	rep320	rep320
Method	cheby2	cheby2	cheby2	cheby2
Line broadening<br>Gaussian/Lorentzian weighting function	1300<br>0.7	1300<br>0.7	1300<br>0.7	1300<br>0.7
Zero filling	32768	32768	32768	32768

Table S2: Additional DFT outputs relative to ^{17}O parameters and ^{7}Li, ^{93}Nb and ^{17}O of Li_3NbO_4.

**$LiNbO_3$ (8x8x4) (ICSD: 84578)**					
	δ_{iso} / ppm	δ_{aniso} / ppm	η_{cs}	C_Q / MHz	η_Q
^{17}O	449	407	0.3	1.1	0.74
**$LiNb_3O_8$ (8x6x3) (ICSD: 2921)**					
	δ_{iso} / ppm	δ_{aniso} / ppm	η_{cs}	C_Q / MHz	η_Q
$^{17}O(1)$	510	298	0.8	1.00	0.6
$^{17}O(2)$	538	490	0.1	0.60	0.6
$^{17}O(3)$	504	526	0.2	-1.25	0.4
$^{17}O(4)$	526	434	0.2	-1.40	0.7
$^{17}O(5)$	406	261	0.8	1.79	0.5
$^{17}O(6)$	519	560	0.3	1.27	0.2
$^{17}O(7)$	510	-307	0.9	0.94	0.7
$^{17}O(8)$	432	345	0.6	1.34	0.4
**Li_3NbO_4 (6x6x6) (ICSD: 243922)**					
	δ_{iso} / ppm	δ_{aniso} / ppm	η_{cs}	C_Q / MHz	η_Q
^{7}Li	0.7 ± 0.5	2.2 ± 0.5	0.6 ± 0.1	-0.04 ± 0.01	0.5 ± 0.1
^{93}Nb	-980 ± 10	-191 ± 5	0.0 ± 0.1	-13 ± 1	0.0 ± 0.1
$^{17}O(1)$	377 ± 5	408 ± 5	0.2 ± 0.1	1.10 ± 0.05	0.0 ± 0.1
$^{17}O(2)$	198 ± 5	8 ± 5	0.0 ± 0.1	0.30 ± 0.05	0.0 ± 0.1

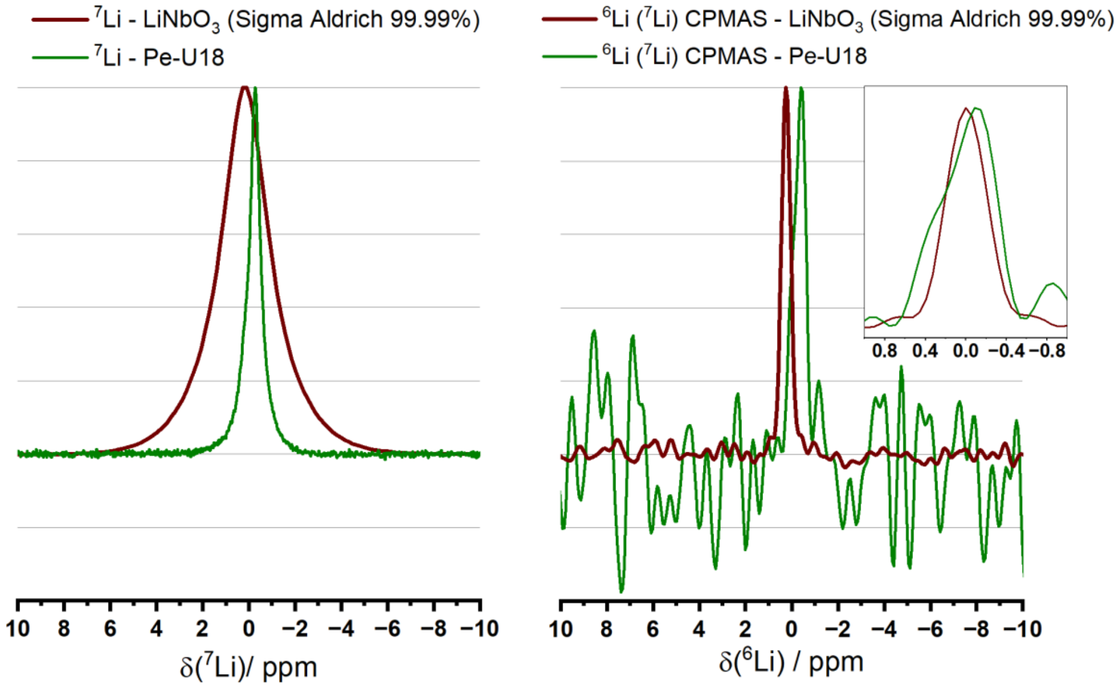

Figure S5: Comparison of the central peak of ^{7}Li MAS spectra (right) the $^{6}Li(^{7}Li)$ CPMAS spectra (left) of LiNbO3 and the Pe-U18 sample shows the different contributions to line broadening. In ^{7}Li MAS, dipolar interactions are not fully averaged, leading to broader lines in samples with shorter Li–Li distances. In contrast, in $^{6}Li(^{7}Li)$ CPMAS, the low natural abundance of ^{6}Li and the ^{7}Li decoupling suppress dipolar interactions. With dipolar largely removed and tine quadrupolar contributions, the observed lineshape mainly reflects the distribution of chemical shifts. The figure illustrates this distinction: for ^{7}Li, pure $LiNbO_3$ displays broadening due to dipolar interactions, whereas the sharp ^{6}Li lines exclude the presence of distinct chemical environments. The difference in signal-to-noise ratio in

the CPMAS spectra is attributed both to the 0.18 times smaller amount of $LiNbO_3$ in Pe-U18 and to the smaller total sample mass available. The weighted sample masses were 0.1487 g for $LiNbO_3$ and 0.0178 g for Pe-U18.

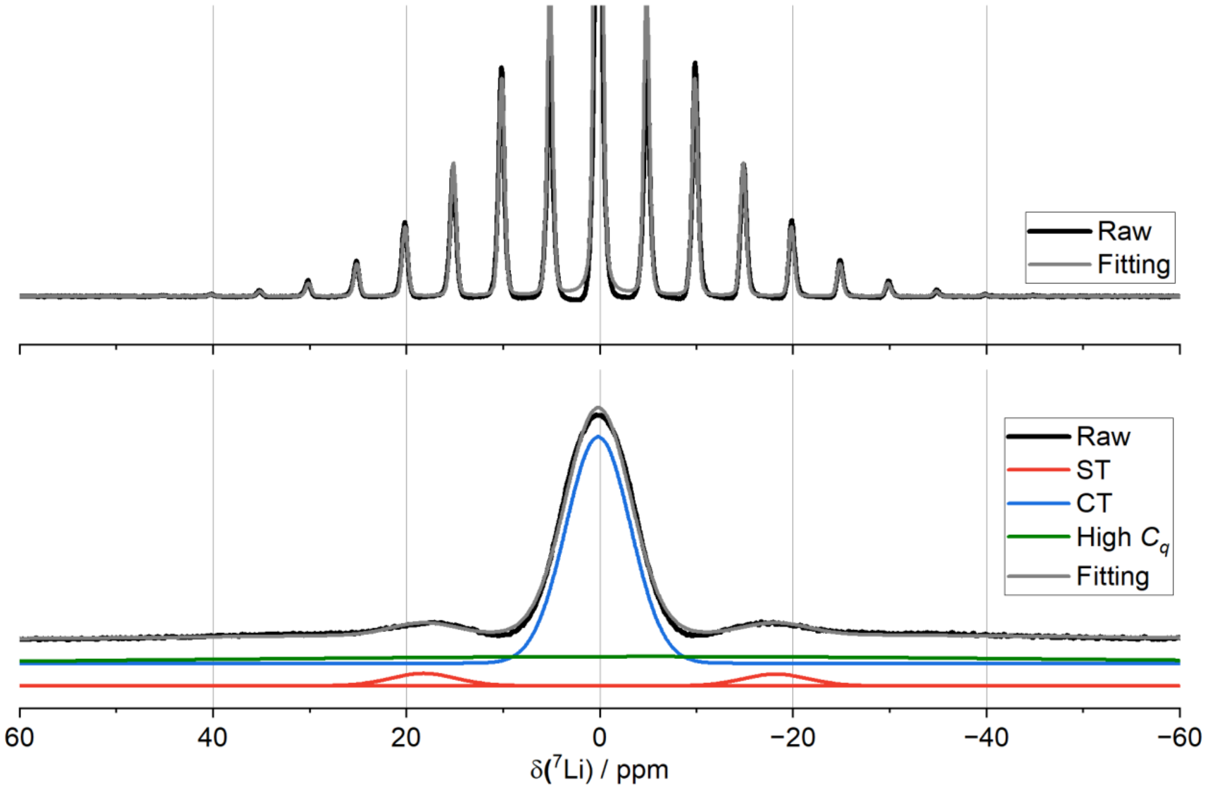

Figure S6: At the top: in black, the ^{7}Li NMR spectrum of $LiNbO_3$ under 5 kHz MAS, and in gray, the corresponding simulation. At the bottom: in black, the ^{7}Li NMR spectrum of $LiNbO_3$ under static conditions. The red lines represent the two satellite transitions, while the blue line corresponds to the central transition. The green line shows a broadened lineshape, possibly caused by defects with stronger quadrupolar interactions. The simulated $\langle C_Q \rangle$ values are 48 kHz for MAS and 36 kHz for the static spectrum. In the static spectra, the $\langle C_Q \rangle$ fitting accounts for the difference in the positions of the two satellite peaks, whereas in the MAS spectra, the contribution from spinning sideband (SSB) intensity also includes defects associated with high-C_Q values. as it allows for the simulation of the two satellite peaks that are characteristic of the $LiNbO_3$ spectrum. In contrast, the MAS spectrum also simulates the component associated with the "high C_q" region observed in the static spectrum, leading to an overestimation of $\langle C_Q \rangle$ in $LiNbO_3$.
